# A Single-Cell Atlas of Transcriptome Changes in the Intestinal Epithelium at the Suckling-to-Weaning Transition in Male Rabbits

**DOI:** 10.1016/j.jcmgh.2025.101628

**Published:** 2025-09-02

**Authors:** Tania Malonga, Christelle Knudsen, Julie Alberge, Emeline Lhuillier, Patrick Aymard, Elisabeth Jones, Corinne Lencina, Manon Despeyroux, Elodie Riant, Cédric Cabau, Alyssa Ivy, Crystal L. Loving, Nathalie Vialaneix, Martin Beaumont

**Affiliations:** 1 GenPhySE, Université de Toulouse, INRAE, ENVT, 31326, Castanet-Tolosan, France; 2 Université de Toulouse, INRAE, UR MIAT, 31326, Castanet-Tolosan, France; 3 GeT-Sante, I2MC, INSERM-Université Paul Sabatier, Plateforme Génome et Transcriptome, GenoToul, Toulouse, France; 4 Plateau de cytométrie I2MC, INSERM, TRI-GenoToul, Université Paul Sabatier, Toulouse, France; 5 Sigenae, GenPhySE, Université de Toulouse, INRAE, ENVT, Castanet-Tolosan, France; 6 Food Safety and Enteric Pathogens Research Unit, National Animal Disease Center, Agricultural Research Service, U.S. Department of Agriculture, Ames, Iowa; 7 Université de Toulouse, INRAE, BioinfOmics, GenoToul Bioinformatics Facility, Castanet-Tolosan, France

**Keywords:** Weaning, Epithelium, Gut, Postnatal Development, Early Life, scRNA-seq, Organoids

## Abstract

**Background & Aims:**

The suckling-to-weaning dietary transition is a key step in intestinal development. The aim of our study was to identify the transcriptome changes induced in each cell type of the intestinal epithelium at the onset of solid food ingestion.

**Methods:**

We compared the single-cell transcriptome of epithelial cells isolated from the cecum of age-matched littermate suckling male rabbits ingesting or not solid food.

**Results:**

Our dataset provides the first single-cell atlas of the rabbit intestinal epithelium and highlights the interest of the rabbit as a model for studying BEST4^+^ epithelial cells, which are absent in mice. Solid food ingestion induced extensive transcriptome changes in each epithelial cell type, with the most pronounced changes noted in absorptive and BEST4^+^ cells. Some of the effects of solid food introduction were common to most epithelial cell types, such as the upregulation of *ALDH1A1*. Solid food ingestion remodeled epithelial defense systems, as observed by the increased expression of interferon-stimulated genes in mature absorptive and BEST4^+^ cells. Solid food upregulated the gene expression of the immunoglobulin transporter *PIGR* in cells located at the base of epithelial crypts and in goblet cells. In addition, solid food triggered epithelial differentiation, which was associated with modification of the expression of genes involved in handling of nutrients, as well as changes in hormone expression by enteroendocrine cells. These cell type–specific transcriptome modifications induced by solid food ingestion coincided with changes in microbiota composition and were replicated, in part, by butyrate in organoids.

**Conclusions:**

Our work provides a single-cell atlas of the transcriptome changes induced in the intestinal epithelium at the suckling-to-weaning transition.

## Graphical abstract

**Figure:**
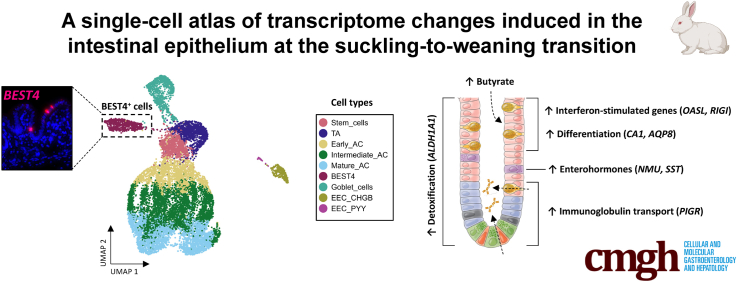

**Summary**

We provide a single-cell atlas of the intestinal epithelium during weaning. Solid food induced extensive, cell type–specific transcriptome changes. BEST4^+^ cells, which are absent in mice, show pronounced responses, highlighting the rabbit as a valuable model to study the mammalian intestinal epithelium.

This article has an accompanying editorial.

The intestinal epithelium contributes to digestion and allows nutrient absorption while providing a physical and immunological barrier against microorganisms and toxic compounds.^1^ This dual functionality is enabled by specialized absorptive and secretory epithelial cells, all derived from actively dividing stem cells located at the crypt base.^2^ Single-cell transcriptomics (single-cell RNA sequencing [scRNA-seq]) has recently deepened our understanding of the cellular diversity of the intestinal epithelium.^3^, ^4^, ^5^ Absorptive cells (ACs) now emerge as a heterogeneous population with distinct functions along the crypt-villus axis.^6^^,^^7^ Additionally, BEST4^+^ cells were recently identified as a novel subset of mature ACs with potential roles in ion transport, mucus hydration, and secretion of antimicrobial peptides and hormones.^8^ Broad cellular diversity is also observed in the secretory cell lineage, as exemplified by the numerous subtypes of enteroendocrine cells (EECs) defined by their hormone secretion profiles.^9^ Heterogeneous populations of mucus-secreting goblet cells have also been identified along the crypt-villus axis with a zonation of their antimicrobial activities.^7^^,^^10^ However, this newly uncovered diversity of epithelial cells is minimally understood in the context of postnatal intestinal development.

After birth, the intestinal epithelium of the mammalian newborn undergoes a maturation process that culminates at the suckling-to-weaning transition.^11^^,^^12^ Indeed, the dietary switch from maternal milk to solid food is associated with an adaptation of epithelial digestion, absorption and transport systems enabling the transition from a high-fat diet to a carbohydrate-based diet.^13^^,^^14^ The onset of solid food ingestion also coincides with a reduced epithelial permeability linked to a remodeling of epithelial defense systems including tight junctions, microbial detection systems, glycosylation, and secretion of antimicrobial peptides and mucus.^15^, ^16^, ^17^, ^18^, ^19^ This epithelial developmental process follows a genetically wired program tuned by several factors including changes in glucocorticoid levels, the introduction of solid food, and the cessation of suckling.^11^^,^^20^, ^21^, ^22^ In addition, ingestion of solid food also strongly alters the composition of the gut microbiota, which contributes to the induction of epithelial maturation, notably through the release of bacterial metabolites such as butyrate.^23^^,^^24^ A defect in host-microbiota co-maturation around weaning is known to increase the susceptibility to inflammatory or metabolic diseases later in life.^25^^,^^26^ It is therefore essential to understand the mechanisms underlying epithelial maturation at the suckling-to-weaning transition. However, a large knowledge gap exists regarding epithelial cell type–specific adaptations triggered by the introduction of solid food in the diet.

Transcriptomic regulations induced in the intestinal epithelium during the transition from milk to solid food were described in mice.^23^^,^^27^ However, these studies did not delineate the relative contributions of age and nutrition in epithelial maturation. In fact, controlling dietary intake in early life is difficult in mice because the stress of separation from the mother disrupts the gut barrier function.^28^ In contrast, in rabbits, suckling occurs only once per day for about 5 minutes and there is naturally little contact between the mother and her litter.^29^ This unique behavior allows to control milk and solid food ingestion in early life by housing separately the mother and her litter.^21^ In addition, we have recently shown that BEST4^+^ cells are present in the intestinal epithelium of rabbits, whereas these cells are absent in mice.^8^ Thus, the rabbit is a valuable model to study the maturation of BEST4^+^ cells at the suckling-to-weaning transition. In this study, we used single-cell transcriptomics to identify the maturation program induced by solid food ingestion in each cell type of the intestinal epithelium of age-matched, suckling, male rabbit littermates fed or not with solid food. In addition, analysis of the microbiota and metabolome allowed us to link the changes in luminal environment induced by solid food ingestion with the gene expression regulation observed at the single-cell level in the epithelium.

## Results

**Figure 1:**
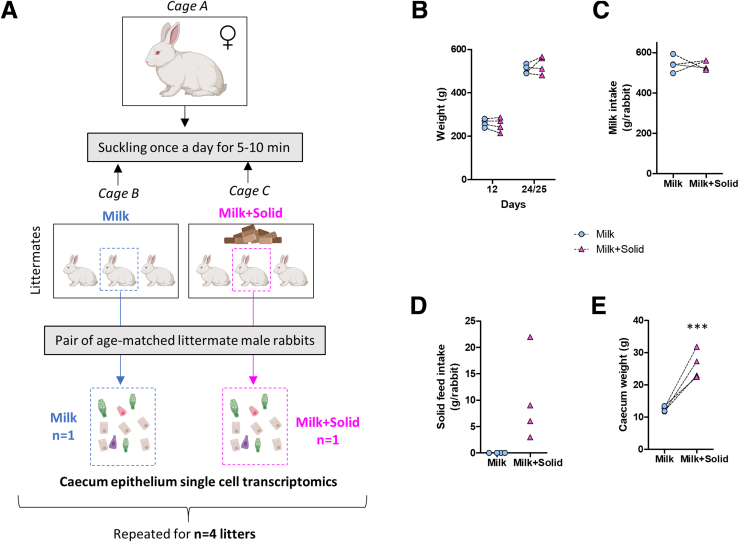
**Experimental design.** (*A*) Schematic representation of the experimental design that was repeated for 4 litters. At PND12, pups within each litter were separated in 2 cages (3 pups/cage) adjacent to the cage of their mother to form 2 groups. In the first group (milk), the pups were exclusively suckling. In the second group (milk+solid), the pups were suckling while having access to solid food. The dam and the pups were regrouped in a nest once a day for 5–10 minutes for suckling before returning to their respective cages. On PND24/25, 1 pup per litter from each group was sacrificed for the isolation of cecal epithelial cells and single-cell RNA-sequencing (milk group: n = 4 pups, milk+solid group: n = 4 pups). (*B*) Rabbit weights at days 12 and 24/25. (*C*) Total milk intake per rabbit between days 12 and 24/25. (*D*) Total solid feed intake per rabbit between days 12 and 24/25. Feed intake was estimated at the cage level (3 pups) by weighing the feeder. Data points represent values measured in each cage. (*E*) Full cecum weight per rabbit. ∗∗∗*P < .*001. (*B, C, E*) Points represent individual values in rabbits and dotted lines link littermates.

To evaluate the effects of solid food introduction on epithelial maturation, we determined single-cell transcriptomic profiles of cecal epithelial cells isolated from 4 pairs of age-matched littermate suckling rabbits ingesting or not solid food (milk group: n = 4, milk+solid group: n = 4) (Figure 1*A*). Growth and milk intake were similar in the 2 groups (Figure 1*B* and *C*). In the milk+solid group, the small amount of solid food ingested (<25 g/day/rabbit) increased dramatically the weight of the cecum (Figure 1*D* and *E*).

### A Single-Cell Atlas of the Rabbit Gut Epithelium

**Figure 2:**
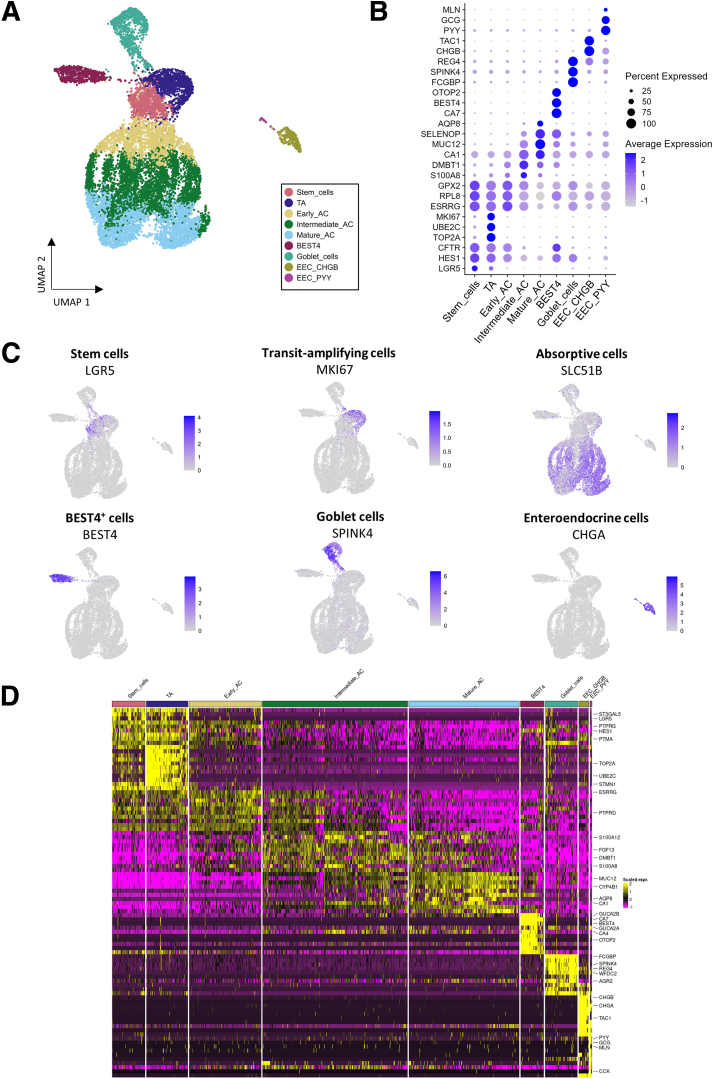
**A single-cell transcriptomic atlas of the rabbit cecum epithelium.** (*A*) UMAP of cells colored by epithelial cell type. The 13,805 cells were derived from 8 suckling rabbits ingesting or not solid food (n = 4 per group). (*B*) Expression of selected marker genes for each cell type (average expression across cells in color and percentage of cells expressing the marker in size). (*C*) UMAPs colored by the expression of marker genes of each cell type. (*D*) Average expression of the top 10 marker genes with the highest average log2(fold change) for each cell type. For a given cell type, markers were ordered by decreasing log2(fold change) of the expression between this cell type and the other types.

After applying quality filters and removal of hematopoietic cells, the dataset included 13,805 cecal epithelial cells. We identified 13 clusters of cells based on their transcriptome profiles. Each cluster was assigned to a cell type based on expression of known markers of epithelial lineages (Figure 2*A–D*; Supplementary Table 1).

**Figure 3:**
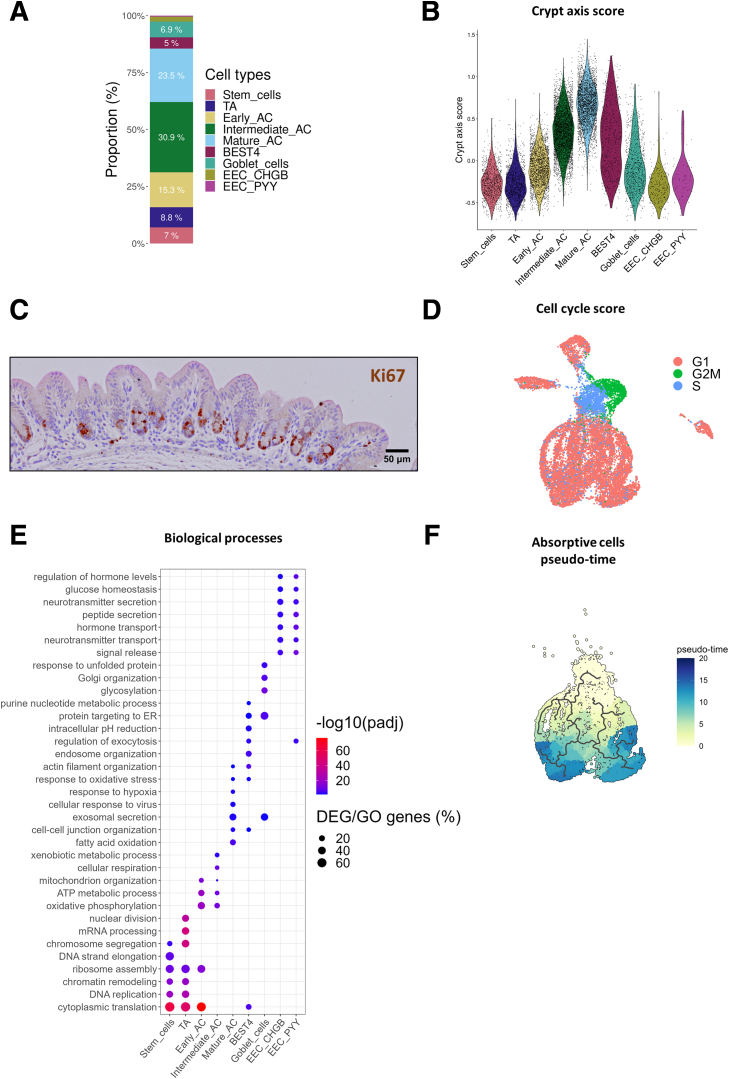
**Transcriptionally distinct cell populations in the rabbit cecum epithelium.** (*A*) Relative abundance for each cell type. (*B*) Crypt axis score for each cell type. (*C*) Localization of transit amplifying cells in the rabbit cecum epithelium by immunostaining of Ki67 (brown). Nuclei are stained in blue. Scale bar 50 μm. (*D*) UMAP colored by the inferred cell cycle state. (*E*) Selected biological processes enriched in marker genes for each cell type. The color corresponds to the –log10(adjusted *P* value) of the overrepresentation test and the size corresponds to the percentage of marker genes among the genes of the ontology term. (*F*) UMAP colored by the pseudo-time of the stem and ACs.

Stem cells (*LGR5*^+^; 7% of epithelial cells) and transit-amplifying (TA) cells (*MKI67*^*+*^, *TOP2A*^*+*^, *UBE2C*^+^; 8.8% of epithelial cells) were predicted to be positioned at the crypt base (Figures 2 and 3*A* and *B*; Supplementary Table 1). Immunodetection of Ki67 (encoded by *MKI67*) confirmed that TA cells were localized at the crypt base (Figure 3*C*). Stem and TA cells were predicted in the S and G2M cell cycle phases, respectively, while most other epithelial cells were in the G1 phase (Figure 3*D*). Both stem and TA cells expressed high levels of genes involved in DNA replication and translation (Figure 3*E*; Supplementary Table 2). TA cells specifically expressed genes related to messenger RNA (mRNA) processing and nuclear division (Figure 3*E*; Supplementary Table 2). ACs (*SLC51B*^+^) were the main epithelial cell population and the distribution of their pseudo-times indicated 3 main differentiation states that we termed as early (15.3% of epithelial cells), intermediate (30.9% of epithelial cells) and mature (23.5% of epithelial cells) (Figures 2*A–C* and 3*A* and *F*). AC gene expression profiles showed gradual modifications according to their differentiation states, which reflect their maturation during migration along the crypt axis (Figure 2*D*). Indeed, early ACs were predicted to be localized at the lower part of crypts, while mature ACs were positioned at the crypt top (Figure 3*B*). Gene expression profile of early ACs was transitional between stem/TA cells and other ACs (Figure 2*B* and *D*). Intermediate ACs expressed high levels of genes involved in antimicrobial defenses (eg, *S100A8*, *S100A12*, *DMBT1*) and in mitochondrial metabolism (Figures 2*B* and *D* and 3*E*; Supplementary Tables 1 and 2). Mature ACs highly expressed genes involved in epithelial digestion, transport, and glycocalyx formation (eg, *CA1*, *AQP8*, *ABCA1*, *APOB*, *ANPEP*, *MUC12*) (Figure 2*B* and *D*; Supplementary Table 1), and genes involved in lipid metabolism, response to hypoxia, and antimicrobial defenses (Figure 3*E*; Supplementary Table 2).

**Figure 4:**
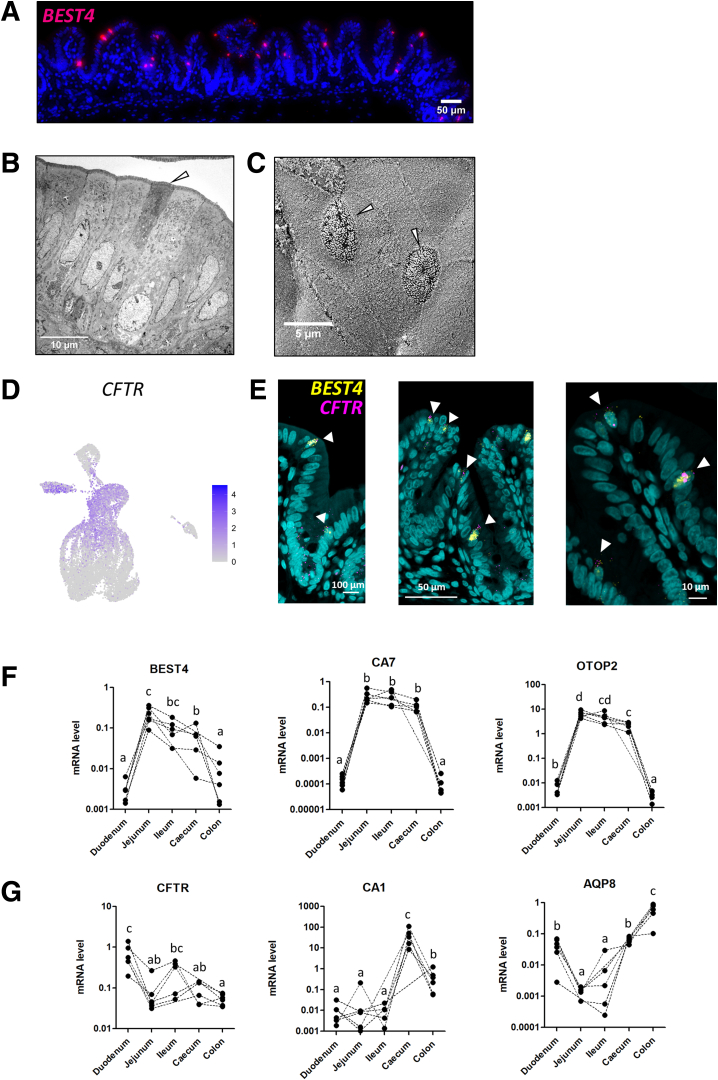
**BEST4^+^ cells in the rabbit intestine.** (*A*) Localization of BEST4^+^ cells in the rabbit cecum epithelium by in situ hybridization of *BEST4* mRNA (red). Nuclei are stained in blue. Scale bar 50 μm. (*B*) Transmission electron microscopy observation of ACs. The white arrowhead shows an electron dense AC. Scale bar 10 μm. (*C*) Scanning electron microscopy observation of microvilli. White arrowheads show cells with low density of microvilli. Scale bar 5 μm. (*D*) UMAP of cells colored by the expression of *CFTR*. (*E*) Dual in situ hybridization of *CFTR* mRNA (pink) and *BEST4* mRNA (yellow) in rabbit cecum epithelium. Scale bars 100 μm (left), 50 μm (middle), and 10 μm (right). Nuclei are stained in blue. White arrowheads show cell stained with both *CFTR* and *BEST4*. (*F*) Gene expression of *BEST4*, *CA7*, and *OTOP2* in tissue sections of duodenum, jejunum, ileum, cecum, and colon. (*G*) Gene expression of *CFTR*, *CA1*, and *AQP8* in tissue sections of duodenum, jejunum, ileum, cecum, and colon. (*F, G*) Points represent individual values in rabbits and dotted lines link intestinal region from the same rabbit. Expression values in intestinal regions associated with different letters are significantly different (*P* < .05).

BEST4^+^ cells (5% of epithelial cells), a recently discovered subset of mature absorptive epithelial cells, were identified by the expression of the canonical markers *BEST4*, *CA7*, *OTOP2*, *GUCA2B*, *GUCA2A*, and *CFTR* (Figures 2*A–D* and 3*A*; Supplementary Table 1). BEST4^+^ cells were predicted to be distributed along the crypt axis (Figure 3B). *BEST4* mRNA in situ hybridization confirmed that BEST4^+^ cells are relatively rare (<4 BEST4^+^ cells per crypt) and distributed along the crypt axis (Figure 4*A*). Functions enriched in BEST4^+^ cells included “regulation of exocytosis” and “intracellular pH reduction” (Figure 3*E*; Supplementary Table 2). Although the morphological features of BEST4^+^ cells are not defined yet, we observed rare electron dense ACs and cells with low-density microvilli that may correspond to distinct subsets of absorptive epithelial cells (Figure 4*B* and *C*). Dual mRNA in situ hybridization of *CFTR* and *BEST4* confirmed that *CFTR* is expressed by BEST4^+^ cells in the rabbit cecum epithelium (Figures 2*B* and 4*D* and *E*). *CFTR* mRNA was also detected at the base of epithelial crypts, which is consistent with our scRNA-seq data showing the expression of *CFTR* in stem cells, TA cells, and early ACs (Figures 2*B* and 4*D* and *E*). Given that *CFTR* expression was previously found to be restricted to BEST4^+^ cells in the human small intestine,^5^ we analyzed the expression of BEST4^+^ cell markers in tissue sections collected along the rabbit small and large intestine. We found that the expression of *BEST4*, *CA7*, and *OTOP2* was higher in the jejunum, ileum, and cecum than in the duodenum and colon (Figure 4*F*). *CFTR* expression did not mirror the expression of BEST4^+^ cell markers as *CFTR* expression was the highest in the duodenum mucosa (Figure 4*G*). The high expression of *CA1* in the cecum and of *AQP8* in the colon confirmed the expected patterns of regional gene expression in the large intestine (Figure 4*G*).

**Figure 5:**
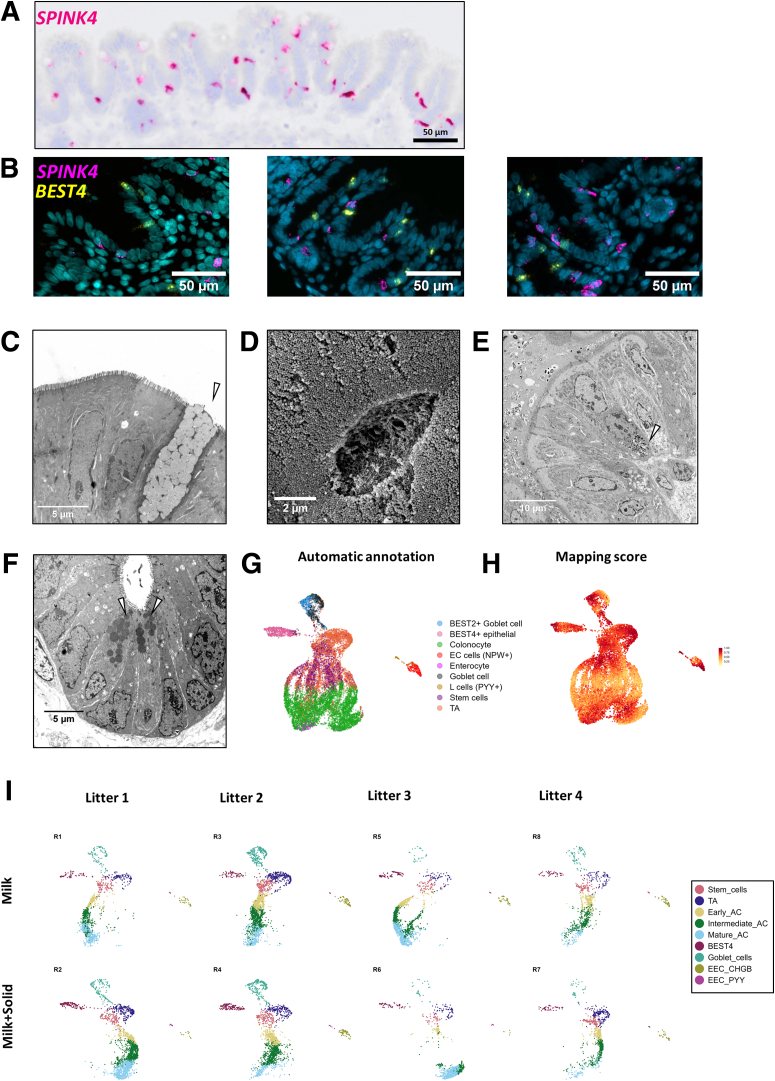
**Secretory cells in the rabbit cecum epithelium and homology with human epithelial cells.** (*A*) Localization of goblet cells in the rabbit cecum epithelium by in situ hybridization of *SPINK4* mRNA (red). Nuclei are stained in blue. Scale bar 50 μm. (*B*) Localization of goblet cells and BEST4^+^ cells in cecum epithelial crypts of rabbits by dual in situ hybridization of *SPINK4* mRNA (pink) and *BEST4* mRNA (yellow). Nuclei are stained in blue. Scale bar 50 μm. (*C*) Transmission electron microscopy (TEM) observation of a goblet cell. The white arrowhead shows mucin granules. Scale bar 5 μm. (*D*) Scanning electron microscopy observation of a goblet cell. Scale bar 2 μm. (*E*) TEM observation of an enteroendocrine cell containing basal electron dense granules (white arrowhead). Scale bar 10 μm. (*F*) TEM observation of Paneth-like cells containing apical electron dense granules (white arrowheads) at the crypt base. Scale bar 5 μm. (*G*) UMAP colored by label transfer from human large intestine epithelial cells to rabbit cecal epithelial cells. (*H*) UMAP colored by mapping score calculated using the large intestine human epithelium as a reference. (*I*) UMAP colored by epithelial cell types for each rabbit order by groups (rows) and litters (columns). EC, enterochromaffin cell.

Goblet cells (6.9% of epithelial cells) were identified by expression of known markers of this lineage and of major components of mucus (*SPINK4*, *REG4*, *FCGBP*, *WFDC2*, *AGR2*, *ZG16*, *TFF3*) (Figures 2*B–D* and 3*A*; Supplementary Table 1). Goblet cells were predicted to be distributed across the crypt axis (Figure 3*B*), and we confirmed their localization by *SPINK4* mRNA in situ hybridization (Figure 5*A*). Dual staining confirmed that *SPINK4* and *BEST4* mRNA were expressed by distinct epithelial cells (Figure 5*B*). Genes specifically expressed by goblet cells were involved in “glycosylation” and “Golgi organization” (Figure 3E; Supplementary Table 2), which reflects their role in the synthesis of mucins, also illustrated by goblet cells morphological features (Figure 5*C* and *D*). Enteroendocrine cells (*CHGA*^*+*^, *NEUROD1*^+^) specifically expressed genes involved in the secretion of hormones (Figures 2*B–D* and 3E; Supplementary Tables 1 and 2). Two subclusters of EECs were distinguished based on their repertoire of hormone-related genes. EEC CHGB^+^ (2.2% of epithelial cells, enterochromaffin-like cells) expressed *CHGB*, *TAC1*, *TTR*, *NMU*, and *TPH1* whereas EEC PYY^+^ (0.4% of epithelial cells, L-like cells) expressed *PYY*, *GCG*, *MLN*, and *CCK* (Figures 2*B* and *D* and 3*A*; Supplementary Table 1). Electron microscopy confirmed the presence of rare enteroendocrine cells containing electron dense granules at the basal side (Figure 5*E*).

Other rare cell types described in the intestinal epithelium of other species (tuft cells, Paneth cells, M cells) were not found in our rabbit cecum epithelium scRNA-seq dataset. However, we observed at the base of epithelial crypts a few Paneth-like cells containing electron-dense apical granules, whose scarcity may preclude their capture in droplets (Figure 5*F*). Automatic cell annotation based on human large intestine scRNA-seq data was consistent with the manual assignment of cell types (Figure 5*G*). The mapping score indicating the degree of similarity between rabbit and human cells was highest for stem cells, TA cells, BEST4^+^ cells, mature ACs, and subsets of goblet and enteroendocrine cells, while the lowest similarity was observed in intermediate ACs (Figure 5*H*). All cell types were identified in each rabbit (Figure 5*I*). In sum, our analysis provided the first single-cell transcriptomic atlas of the rabbit intestinal epithelium. We have made these gene expression data available as a searchable tool on the Broad Institute Single Cell Portal (https://singlecell.broadinstitute.org/single_cell/study/SCP2662/single-cell-transcriptomics-in-caecum-epithelial-cells-of-suckling-rabbits-with-or-without-access-to-solid-food#study-visualize).

### Solid Food Introduction Induced Both Global and Cell Type–Specific Transcriptomic Modifications in the Intestinal Epithelium

**Figure 6:**
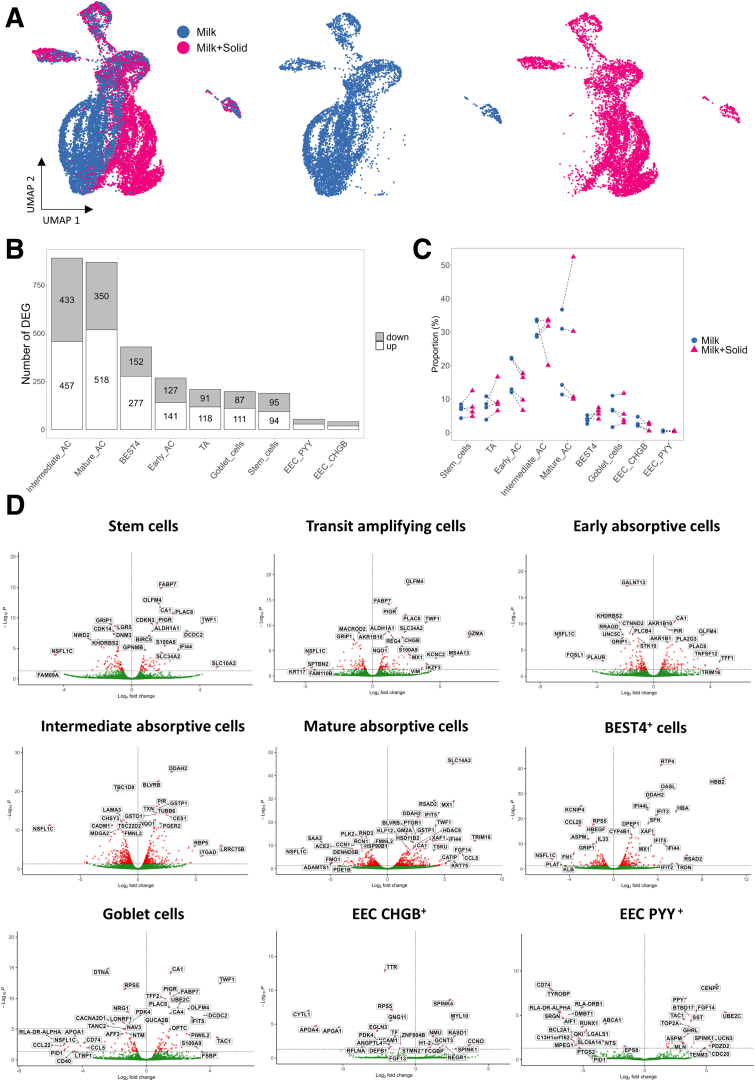
**Ingestion of solid food by suckling rabbits modulates the transcriptome of each epithelial cell type.** (*A*) UMAP of epithelial cells colored by group. (Left) UMAP of merged datasets, with cells restricted to milk (n = 4) (middle) and milk+solid (n = 4) (right) groups. (*B*) Number of DEGs between groups per cell type. Gray bars represent downregulated genes in the milk+solid group while white bars represent upregulated genes in the milk+solid group. DEGs were obtained by using negative binomial generalized linear models on pseudo-bulk data fitted independently in each cell type. (*C*) Relative abundance of each epithelial cell type. Points represent individual values per rabbit and dotted lines link littermates. (*D*) Volcano plots of test results for each cell type. The –log10(adjusted *P* value) are plotted on the y-axis and the log2(fold change) values are plotted on the x-axis.

After characterizing the cellular diversity of the rabbit intestinal epithelium, we focused our analysis on the effects of solid food introduction on gene expression in each epithelial cell type. Ingestion of solid food altered the transcriptome of ACs, as suggested in the Uniform Manifold Approximation and Projection (UMAP) by the low overlap between ACs from suckling rabbits ingesting or not solid food (Figure 6*A*). Accordingly, the highest number of differentially expressed genes (DEGs) was found in intermediate and mature ACs with 890 and 868 DEGs, respectively (Figure 6*B* and *D*). Although to a lesser extent, solid food introduction also modified the transcriptome in BEST4^+^ cells (429 DEGs), early ACs (268 DEGs), TA cells (209 DEGs), goblet cells (198 DEGs), stem cells (189 DEGs), EEC PYY^+^ (54 DEGs), and EEC CHGB^+^ (41 DEGs) (Figure 6*B* and *D*). These solid food–induced alterations of gene expression were observed despite the proportion of epithelial cell types remaining similar in the two groups (Figure 6*C*). The proportion of mature ACs varied greatly between litters. Supplementary Table 3 provides the list of DEGs for each cell type. Supplementary Table 4 contains the results of the enrichment analysis using DEGs of each cell type. All the biological functions and genes cited below were significantly modulated following the introduction of solid food (adjusted *P < .*05).

**Figure 7:**
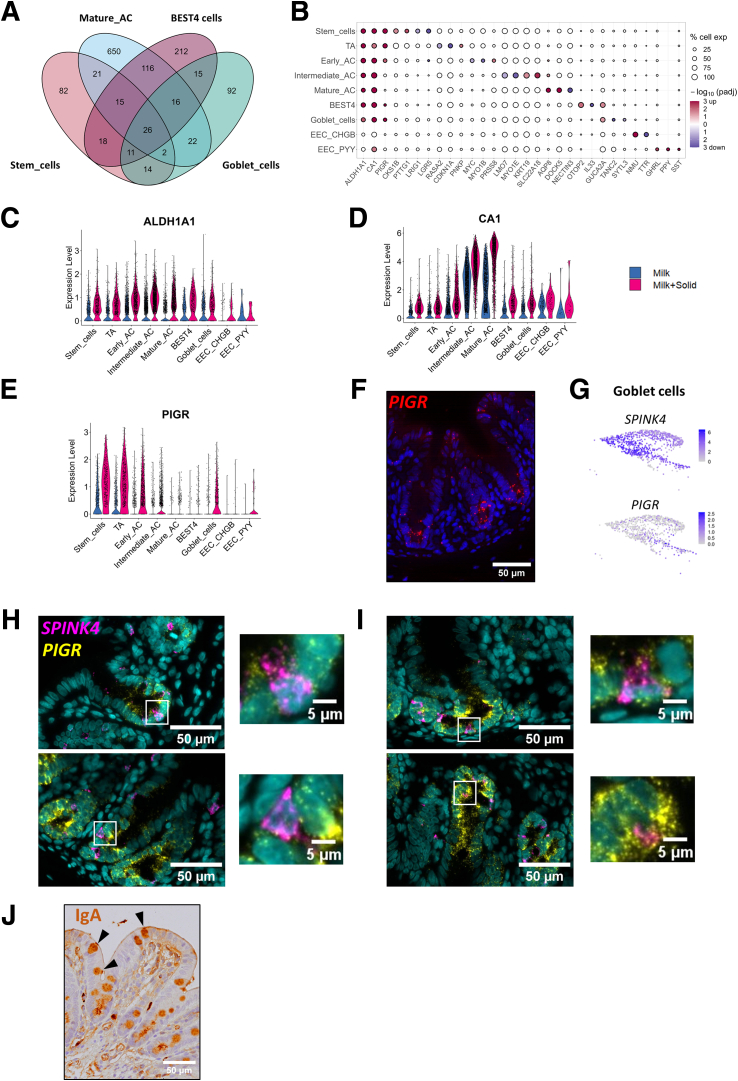
**Transcriptomic changes induced by the introduction of solid food and shared between several cell types.** (*A*) Number of DEGs that are cell type–specific or shared by multiple cell types for stem cells, mature ACs, BEST4^+^ cells, and goblet cells. (*B*) Selected DEG significance (log10(adjusted *P* value), color) and fold change sign (red for over expressed and blue for under expressed in the milk+solid group vs the milk group), by cell type. The size represents the percentage of cells expressing the gene in the corresponding cell type. (*C–E*) Expression level of selected DEGs shared between several cell types, by cell (dot) per cell type and group. (*F*) Localization of *PIGR* mRNA (red) by in situ hybridization in the rabbit cecum epithelium. Nuclei are stained in blue. Scale bar 50 μm. (*G*) UMAP of goblet cells colored by the expression of *SPINK4* or *PIGR*. (*H, I*) Dual in situ hybridization of *SPINK4* mRNA (pink) and *PIGR* mRNA (yellow) in cecum epithelial crypts of rabbits. Nuclei are stained in blue. Scale bars 50 μm (crypts) and 5 μm (insets). (*H*) Observation of *SPINK4*^+^/*PIGR*^–^ cells. (*I*) Observation of *SPINK4*^+^/*PIGR*^+^ cells. (*J*) Immunostaining of IgA (brown) in the rabbit cecum epithelium. Black arrowheads show IgA^+^ cells with a goblet cell morphology. Nuclei are stained in blue. Scale bar 50 μm.

Most of the modifications of gene expression induced by the introduction of solid food were cell type specific while other changes were shared between cell types (Figure 6*D*). Notably, mature ACs and BEST4^+^ cells shared a high number of DEGs (Figure 7*A*). Among transcriptomic modifications shared between most cell types, solid food introduction induced a strong upregulation of *ALDH1A1*, encoding an enzyme involved in retinoic acid metabolism, and an upregulation of *CA1*, a typical marker of epithelial differentiation in the large intestine (Figure 7*B–D*). Solid food introduction also increased the gene expression of the immunoglobulin transporter *PIGR* in several cell types, and this effect was much more pronounced in cells located at the bottom of the crypts (Figure 7*B* and *E*). *PIGR* mRNA in situ hybridization confirmed its predominant expression at the base of epithelial crypts (Figure 7*F*). The expression of *PIGR* was also increased by solid food ingestion in a subset of goblet cells (Figure 7*B* and *E*). Indeed, 34% of *SPINK4*^+^ goblet cells expressed *PIGR* and this observation was supported by dual mRNA in situ hybridization of *SPINK4* and *PIGR* in some goblet cells localized at the base of crypts (Figure 7G–I). Additionally, goblet cells were found to contain immunoglobulin A (IgA) (Figure 7J).

**Figure 8:**
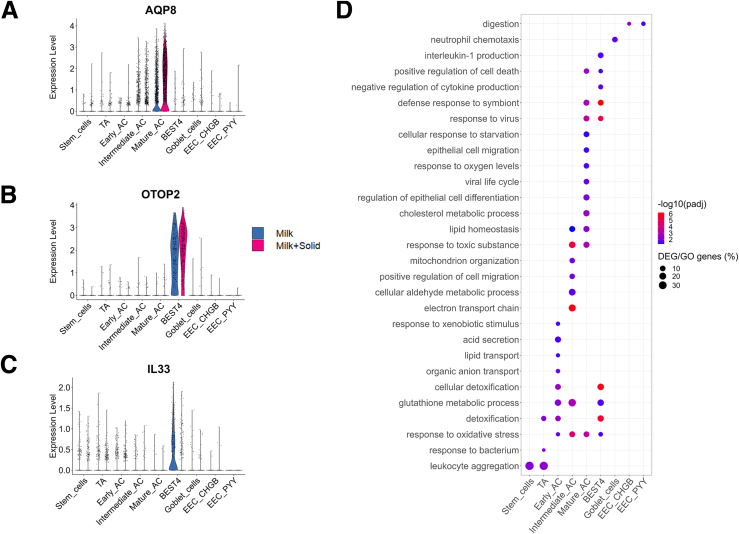
**Cell type–specific transcriptomic changes induced by the introduction of solid food.** (*A–C*) Expression level of selected DEGs specific of a single cell type, by cell (dot), per cell type and group. (*D*) Selected biological processes enriched in DEGs of each cell type. The color corresponds to the –log10(adjusted *P* value) and the size represents the percentage of DEGs included in the biological process.

Among the cell type–specific modifications, solid food ingestion reduced the gene expression of *LRIG1*, a master regulator of the stem cell niche, exclusively in stem cells (Figure 7*B*). Conversely, ingestion of solid food upregulated the gene expression of the transcellular water transporter *AQP8*, mostly in mature ACs (Figures 7*B* and 8*A*). In BEST4^+^ cells, solid food introduction increased the expression of the pH-sensitive ion channel *OTOP2*, while it reduced the expression of the interleukin *IL33* (Figures 7*B* and 8*B* and *C*). Overall, our results showed that the introduction of solid food–induced major transcriptomic modifications in the intestinal epithelium of suckling rabbits, and these changes are either shared across cell types or are cell type specific. Accordingly, enrichment analyses revealed that solid food ingestion altered specific functions in every epithelial cell type (Figure 8*D*; Supplementary Table 4).

### Solid Food Introduction Remodels Defense Systems in the Intestinal Epithelium

**Figure 9:**
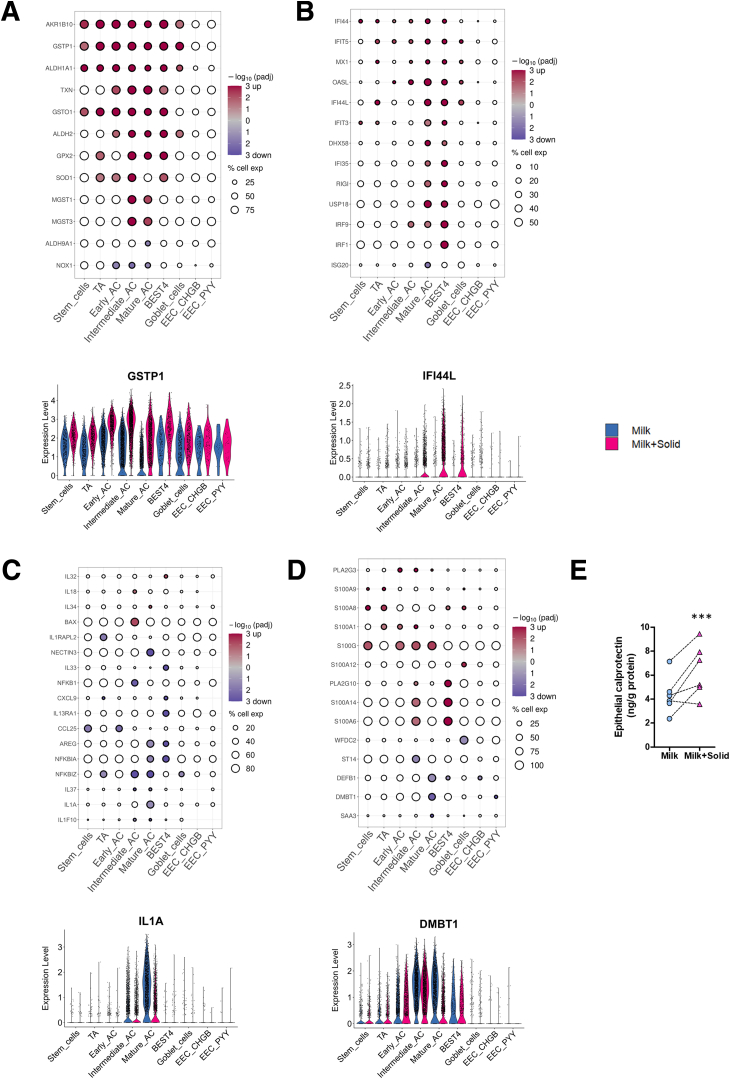
**The introduction of solid food modifies the expression of genes involved in epithelial defenses.** (*A–D*) (Top) Selected DEG significance (log10(adjusted *P* value), color) and fold change sign (red for over expressed and blue for under expressed genes in the milk+solid group vs the milk group) involved in (*A*) detoxification and redox balance, (*B*) interferon signaling, (*C*) cytokine signaling, and (*D*) antimicrobial peptides. The size corresponds to the percentage of cells expressing the gene in the cell type. Bottom: Expression level of a selected DEG by cell (dot), per cell type and group. (*E*) Concentration of calprotectin in cecum epithelial cells. Points represent individual values per rabbit and dotted lines link littermates. ∗∗∗*P < .*001.

Solid food introduction upregulated the expression of genes involved in detoxification in all cell types, except EECs (eg, *GPX2*, *GSTO1*, *GSTP1*, *MGST1*, *MGST3*, *SOD1*, *TXN*) (Figure 9*A*). This was particularly marked in intermediate and mature ACs, and in BEST4^+^ cells. This finding is linked to the enrichment of biological pathways related to “cellular aldehyde metabolic process,” “response to toxic substance,” and “response to oxidative stress” in ACs and in BEST4^+^ cells (Figure 8*D*; Supplementary Table 4). Solid food ingestion also increased the expression of interferon-stimulated genes (ISGs), primarily in mature ACs and BEST4^+^ cells (eg, *DHX58*, *OASL*, *IFIT3*, *IFI35*, *IFI44L*, *IRF9*, *MX1*, *USP18*, *RIGI*) (Figure 9*B*), which is consistent with the specific enrichment of biological pathways such as “response to virus” and “defense response to symbiont” in these cell types (Figure 8*D*; Supplementary Table 4). Conversely, solid food ingestion decreased the expression of several genes coding for regulators of innate immune responses in ACs (eg, *AREG*, *NFKBIA*, *NFKBIZ*) (Figure 9*C*). This was associated with a cell type–specific downregulation of genes coding for cytokines in BEST4^+^ cells (*CXCL9*, *IL13RA1*, *IL33*), which were also characterized by a specific enrichment of the biological pathway “negative regulation of cytokine production” (Figure 8*D*; Supplementary Table 4). Other cell type–specific downregulations of cytokine gene expression induced by solid food ingestion included *IL1A* in mature ACs and *CCL25* in stem and early ACs. In contrast, solid food introduction upregulated the expression of other cytokines expressed by small subsets of absorptive and BEST4^+^ cells (eg, *IL18*, *IL32*, *IL34*) (Figure 9*C*). Solid food reduced the expression of some antimicrobial peptides in mature ACs (*DMBT1* and *DEFB1*) and in goblet cells (*WDFC2*) while increasing the expression of numerous antimicrobial proteins of the S100 family in several cell types (eg, *S100A1, S100A12, S100A14, S100A6*, *S100G*) (Figure 9*D*). Interestingly, genes coding for the 2 subunits of the inflammation marker calprotectin (*S100A8/S100A9*) were upregulated, notably in subsets of stem and TA cells (Figure 9*D*). The increased expression of calprotectin by epithelial cells after the ingestion of solid food was confirmed at the protein level in an independent experiment (Figure 9*E*).

**Figure 10:**
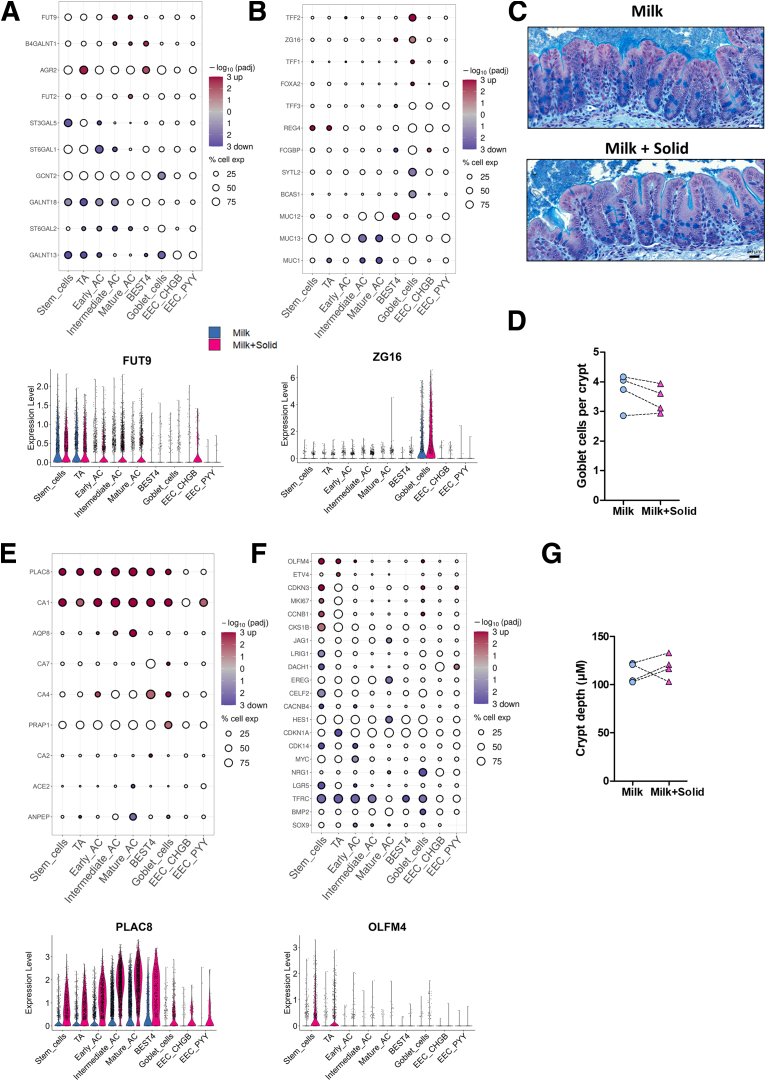
**The introduction of solid food modifies the expression of genes involved in the mucus barrier, epithelial differentiation and renewal.** (*A, B*) (Top) Selected DEG significance (log10(adjusted *P* value), color) and fold change sign (red for over expressed and blue for under expressed genes in the milk+solid group vs the milk group) involved in (*A*) glycosylation and (*B*) mucus components. The size corresponds to the percentage of cells expressing the gene in the cell type. (Bottom) Expression level of a selected DEG by cell (dot), per cell type and group. (*C*) Representative histological observation of the cecum mucosa in each group. Alcian blue shows acidic mucins. Tissues are counterstained with hematoxylin and eosin. Scale bar 20 μm. (*D*) Number of goblet cells per crypt. Points represent individual values per rabbit and dotted lines link littermates. (*E, F*) (Top) Selected DEG significance (log10(adjusted *P* value), color) and fold change sign (red for over expressed and blue for under expressed genes in the milk+solid group vs the milk group) involved in (*E*) differentiation and (*F*) stemness and proliferation. The size corresponds to the percentage of cells expressing the gene in the cell type. (Bottom) Expression level of a selected DEG by cell (dot), per cell type and group. (*G*) Epithelial crypt depth. Points represent individual values per rabbit and dotted lines link littermates.

Solid food introduction also modulated the expression of numerous genes involved in epithelial glycosylation, which plays a key role in host-microbiota interaction. Solid food decreased the expression of several genes coding for glycosyltransferases mostly in stem, TA, early absorptive, and goblet cells (eg, *GALNT13*, *GALNT18 ST3GAL5*, *ST6GAL1*, *ST6GAL2*) while *B4GALT1* was upregulated in mature absorptive and BEST4^+^ cells (Figure 10*A*). In contrast, the introduction of solid food increased the expression of genes coding for fucosyltransferases (*FUT2* and *FUT9*) which are expressed by subsets of ACs (Figure 10*A*). The introduction of solid food also altered the expression of genes related to mucin production (Figure 10*B*). Specifically, solid food ingestion reduced the expression of genes encoding the glycocalyx-forming transmembrane mucins *MUC1* and *MUC13* in intermediate and mature ACs, while enhancing *MUC12* expression in BEST4^+^ cells (Figure 10*B*). In goblet cells, the expression of genes coding for major mucus components were upregulated (eg, *TFF1*, *TFF2*, *ZG16*) or downregulated (eg, *BCAS1*, *SYTL2*) after the introduction of solid food (Figure 10*B*). Histological observations confirmed that the number of goblet cells per crypt was similar in the cecal epithelium of rabbits ingesting or not solid food (Figure 10C and D), which is consistent with the goblet cell proportion estimation obtained by scRNA-seq (Figure 6*C*).

In order to determine whether changes in the expression of genes involved in epithelial defenses were linked to modifications in the microbiota, we performed 16S rRNA gene sequencing in rabbit cecal contents (Supplementary Table 5). Solid food ingestion by suckling rabbits altered the composition of the microbiota, in particular by increasing the abundance of the Lachnospiraceae family (14.6% in the milk group vs 21.2% in the milk+solid group). Altogether, our results show that the introduction of solid food triggered major adaptations of epithelial defense systems in most cell types, which were associated with an alteration of the microbiota composition.

### Solid Food Introduction Enhances Differentiation in Intestinal Epithelial Cells and Alters Nutrient Handling

As a next step, we evaluated how solid food ingestion altered the expression of genes related to epithelial differentiation (Figure 10*E*) and renewal (Figure 10*F*). Key genes involved in stemness and proliferation were upregulated (eg, *CDKN3*, *CKS1B*, *MKI67*, *OLFM4*) or downregulated (eg, *CDK14*, *CELF2*, *DACH1*, *LGR5*, *LRIG1*) in stem cells after the introduction of solid food (Figure 10*F*). These changes at the gene expression level were not associated with a modification of the crypt depth, which is partly determined by epithelial proliferation rate (Figure 10*C* and *G*). In contrast, solid food ingestion strongly increased the expression of the differentiation markers *PLAC8* and *CA1* in most of epithelial cells (Figure 10*E*). Moreover, the solid food–induced upregulation of *AQP8* in mature ACs was associated with the enrichment of the biological pathway “regulation of epithelial cell differentiation” (Figures 8*D* and 10E; Supplementary Table 4). Conversely, in mature ACs, solid food introduction downregulated the expression of *ANPEP*, an enzyme involved in peptide digestion, which is a process usually occurring in the small intestine (Figure 10*E*). Accordingly, automatic annotation of cell types identified a population of enterocyte-like cells in the group of exclusively suckling rabbits (Figures 5*G* and 6*A*).

**Figure 11:**
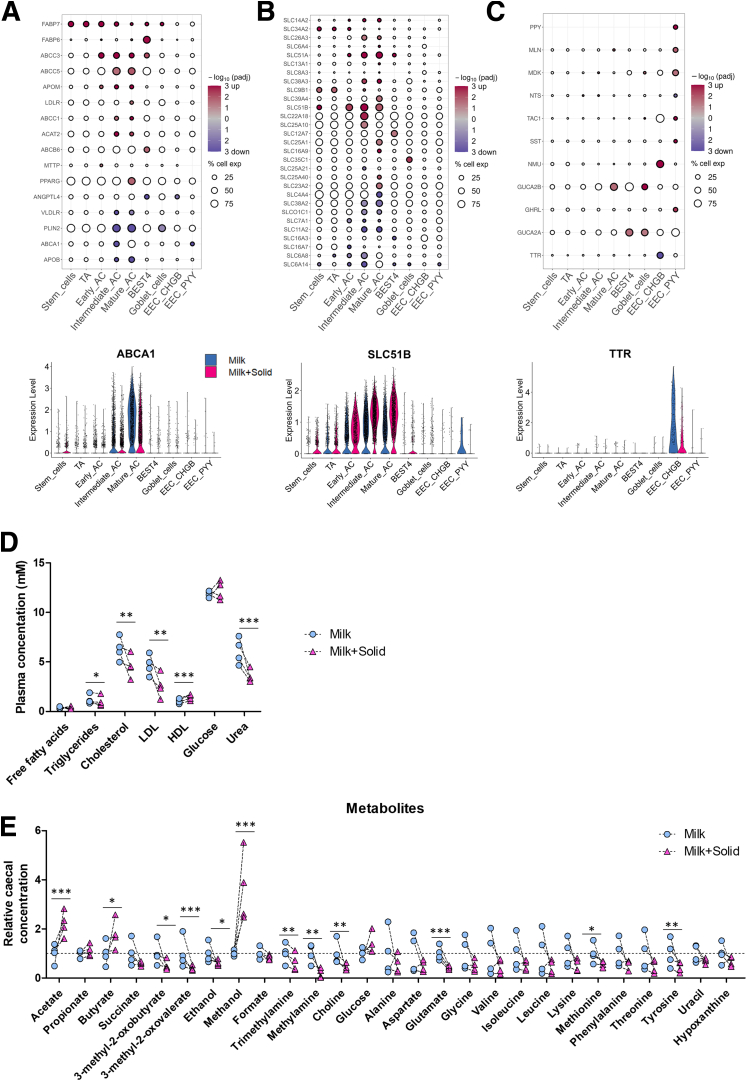
**Solid food–induced modifications of the expression of genes involved in epithelial nutrient handling is associated with changes in concentrations of plasma and cecal metabolites.** (*A–C*) (Top) Selected DEG significance (log10(adjusted *P* value), color) and fold change sign (red for over expressed and blue for under expressed genes in the milk+solid group vs the milk group) involved in (*A*) lipid metabolism, (*B*) epithelial transport, and (*C*) hormone secretion. The size corresponds to the percentage of cells expressing the gene in the cell type. (Bottom) Expression level of a selected DEG by cell (dot), per cell type and group. (*D*) Plasmatic concentrations of metabolites. Points represent individual values per rabbit and dotted lines link littermates. ∗*P* < .05, ∗∗*P* < .01, ∗∗∗*P* < .001. (*E*) Relative cecal concentrations of metabolites detected by nuclear magnetic resonance–based metabolomics. Points represent individual values per rabbit and dotted lines link littermates. ∗*P* < .05 (adjusted), ∗∗*P* < .01 (adjusted), ∗∗∗*P* < .001 (adjusted). HDL, high-density lipoprotein; LDL, low-density lipoprotein.

These results suggesting a rewiring of AC functions after solid food introduction were associated with the modulation of the expression of numerous genes involved in lipid handling and chylomicron biogenesis, mostly in ACs (Figure 11*A*). These genes were either downregulated (*ABCA1*, *APOB*, *PLIN2*, *VLDR*) or upregulated (*ACAT2*, *APOM*, *LDLR)* (Figure 11*A*). Accordingly, AC DEGs were enriched in functions related to “lipid transport,” “lipid homeostasis,” and “cholesterol metabolic process” (Figure 8*D*; Supplementary Table 4). Moreover, solid food ingestion increased the expression of several bile acid transporters, such as *FABP7* that was upregulated in most cell types, *FABP6* that was specifically upregulated in BEST4^+^ cells, and *SLC51A* and *SLC51B* that were mostly upregulated in ACs (Figure 11*A* and *B*). These alterations of the expression of lipid processing genes were coupled with an important decrease in the plasmatic concentration of cholesterol and low-density lipoprotein after solid food introduction (Figure 11*D*).

In addition, the introduction of solid food altered the expression of numerous genes coding for solute carriers (Figure 11*B*). Ingestion of solid food strongly upregulated the expression of genes coding for the urea transporter *SLC14A2* and several ion transporters (eg, *SLC11A2*, *SLC22A18*, *SLC26A3*, *SLC39A4)* in ACs (Figure 11*B*). The upregulation of the urea transporter coincided with a significant decrease in plasma urea concentration following the introduction of solid food (Figure 11*D*). Solid food ingestion also specifically upregulated the ion transporter *SCL12A7* in BEST4^+^ cells and the fucose transporter *SLC35C1* in goblet cells. The introduction of solid food altered the expression of monocarboxylic acid transporters with an upregulation of *SLC16A9* in mature ACs, a downregulation of *SLC16A3* in BEST4^+^ cells and a downregulation of *SLC16A7* in early ACs (Figure 11*B*). This result can be linked to the increased concentrations of the bacterial short chain fatty acids acetate and butyrate in the cecum content after the introduction of solid food (Figure 11*E*). Similarly, the lower concentration of amino acids (glutamate, methionine, and tyrosine) following solid food introduction can be associated with the upregulation (*SLC38A3)* and downregulation (*SLC38A2*, *SLC6A14*, *SLC7A1*) of amino acids transporters in ACs (Figure 11*B* and *E*).

The solid food–induced alterations of nutrient handling were associated with modifications of gene expression in EECs (Figure 11*C*). In EEC PYY^+^ cells, solid food introduction upregulated the expression of genes coding for the hormones *GHRL*, *MDK*, *MLN*, *SST*, *PPY*, and *TAC1*, while only *NTS* was downregulated. In *EEC CHGB*^*+*^, solid food ingestion increased the expression of the hormone coding gene *NMU*, while *TTR* was downregulated (Figure 11*C*). These observations are reflected by the EEC-specific enrichment of DEGs involved in the biological pathway “digestion” (Figure 8*D*; Supplementary Table 4). In addition, genes coding for the hormones involved in the guanylate cyclase C signaling (*GUGA2A* and *GUCA2B*) were upregulated in goblet cells, mature ACs, and BEST4^+^ cells (Figure 11*C*). Overall, our results show that solid food ingestion enhances epithelial differentiation and remodels the sensing, transport, and metabolism of nutrients by epithelial cells.

### Solid Food–Induced Changes in Gene Expression Are Partly Replicated by Butyrate in Cecum Organoids

**Figure 12:**
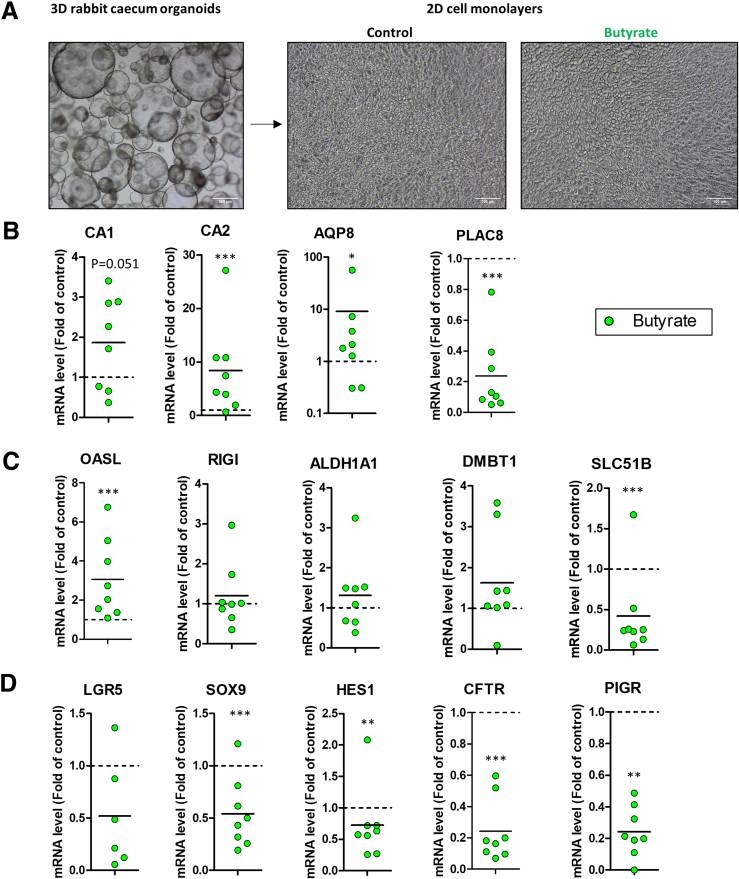
**The gut microbiota–derived metabolite butyrate modifies gene expression in cell monolayers derived from rabbit cecum organoids.** (*A*) Experimental design. Rabbit cecum organoid cell monolayers were treated or not with butyrate (5 mM) for 2 days. (Left) A representative observation of 3D rabbit cecum organoids (scale bar 500 μm). Representative observations of organoid cell monolayers untreated (middle) or treated (right) with butyrate (scale bar 100 μm). (*B–D*) Gene expression in organoid cell monolayers treated by butyrate. Data are expressed relatively to the value measured in the control condition in the same experiment, represented by the dotted line (y = 1). Data points shows values measured in an individual cell culture insert. Horizontal bars show the mean value. Significant differences with the control are indicated by ∗*P < .*05, ∗∗*P < .*01, and ∗∗∗*P < .*001.

We hypothesized that the solid food–induced increased production of butyrate by the gut microbiota (Figure 11*E*) may contribute to the transcriptomic changes observed in the cecum epithelium, as this bacterial metabolite is able to regulate gene expression in host cells.^30^^,^^31^ We therefore analyzed gene expression in rabbit cecum organoid cell monolayers treated or not with 5 mM butyrate on the apical side for 2 days (Figure 12*A*). Butyrate upregulated the gene expression of the differentiation markers *CA2* and *AQP8* (Figure 12*B*), which were also upregulated in vivo after the introduction of solid food in BEST4^+^ cells and in ACs, respectively (Figure 10*E*). Butyrate also tended to increase the expression of *CA1*, which was upregulated in most cell types in vivo after solid food ingestion (Figures 10*E* and 12*B*). In contrast, the upregulation of *PLAC8* observed in vivo in most cell types was not reproduced by butyrate, which decreased the expression of this gene in vitro (Figures 10*E* and 12*B*). Butyrate strongly upregulated the ISG *OASL* (Figure 12*C*), which mirrored the effect of solid food ingestion in ACs, BEST4^+^ cells and goblet cells (Figure 9*B*). In contrast, butyrate had no effect on the expression of *RIGI*, *ALDH1A1*, and *DMBT1*, which were up or downregulated in vivo. The expression of the bile acid transporter *SLC51B* was reduced by butyrate in organoid cell monolayers (Figure 12*C*), whereas the opposite was observed in ACs and BEST4^+^ cells after the introduction of solid food in vivo (Figure 11*B*). Butyrate downregulated the expression of the progenitor cell markers *SOX9* and *HES1* (Figure 12*D*), reflecting the in vivo effect of solid food ingestion (Figure 10*F*). In addition, butyrate reduced the expression of *CFTR* and *PIGR* (Figure 12*D*), an effect that could be attributed to a reduction in stem/progenitor cells that highly express these genes in vivo (Figures 2*B* and 7*E*). Taken together, the butyrate-induced changes in gene expression in organoid cell monolayers suggest that the increased production of this bacterial metabolite after solid food ingestion may contribute to some, but not all, of the transcriptomic changes observed in vivo.

## Discussion

Our study provides the first single-cell transcriptomic atlas of the rabbit intestinal epithelium. This dataset expands the characterization of the cellular diversity of the intestinal epithelium in mammals and constitutes an important resource for the use of rabbits as a model in gastrointestinal research. Our results notably highlighted the diversity of absorptive epithelial cells in the cecum. Indeed, we observed a functional specialization of AC subsets along the cecal crypt axis that mirrored previous findings in the small intestine villi of mice and humans.^6^^,^^7^ For instance, our results showing that middle-crypt ACs specifically express genes coding for antimicrobial peptides is consistent with the observation made in bottom villus enterocytes.^6^^,^^7^ Another important finding of our study is the high homology between rabbit and human BEST4^+^ cells, which indicates that the rabbit is an appropriate animal model to study the role of this subset of mature ACs that are absent in mice.^8^ Our results indicating that rabbit BEST4^+^ cells are mainly localized in the jejunum, ileum and cecum are consistent with studies performed in humans.^8^ However, the expression of the Cl^–^/HCO_3_^–^ channel *CFTR* in BEST4^+^ cells in the rabbit cecum epithelium was unexpected as *CFTR* expression was previously considered to be restricted to BEST4^+^ cells localized in the human small intestine.^5^^,^^32^ The expression of *CFTR* in BEST4^+^ cells in the large intestine may have important implications to understand epithelial fluid efflux, regulation of mucus viscosity, and for the management of cystic fibrosis or diarrheal disease.^33^^,^^34^

Additionally, the two subsets of EECs that we identified in the rabbit cecum (EEC PYY^+^, corresponding to L cells expressing *GCG* and *PYY*; EEC CHGB^+^, corresponding to enterochromaffin cells expressing *TPH1*) were highly similar to human EECs, notably because EECs from both species express the hormones *MLN and* *MDK* which are not expressed by mouse EECs.^9^^,^^10^ The absence of M cells in our cecal epithelium dataset was expected as this cell type is known to be present mostly in the small intestine follicle-associated epithelium.^5^^,^^35^ The lack of Paneth and tuft cells in our single-cell survey could be explained by their scarcity in the large intestine, which reduces their probability of capture by droplets in the microfluidic system.^36^ Indeed, our electron microscopy observations revealed a rare population of Paneth-like cells in rabbit cecal epithelial crypts, confirming previous reports.^37^ Sequencing of a larger number of cells and/or analyzing other gut segments would be required to characterize the transcriptome of these rare epithelial cell types in the rabbit model.

The maturation of the mammalian intestinal epithelium at the suckling-to-weaning transition is considered to be largely driven by genetically wired factors, while the contribution of nutritional and microbial signals remains debated.^11^^,^^19^^,^^21^^,^^22^ Our results clearly demonstrate that the introduction of solid food is sufficient to induce major transcriptome modifications in every intestinal epithelial cell type, independently of age-related factors. Importantly, we observed this strong epithelial response to solid food despite the level of milk intake remaining unchanged, which indicates that the loss of milk-derived factors is not mandatory to induce epithelial maturation. Several previous mouse studies described the transcriptomic changes occurring in the intestinal epithelium at the suckling-to-weaning transition,^23^^,^^27^ but to our knowledge, our study is the first to reveal in which cell types these modifications take place. For instance, we newly demonstrated that the upregulation of the immunoglobulin transporter *PIGR* at the onset of solid food ingestion previously described in mice and rabbits^24^^,^^27^^,^^38^ mainly occurs in epithelial cells localized at the crypt base (stem cells, TA cells, and early ACs). This zonated expression of *PIGR* in crypt base cells that we confirmed by RNA in situ hybridization could be explained by the proximity with the underlying IgA secreting plasma cells. IgA secretion by crypt base cells could contribute to protect the stem cell niche from microorganisms. In line with our findings, the compartmentalization of *PIGR* expression in the mouse and human intestinal epithelium was recently demonstrated to be driven by BMP signaling, which increases from the crypt base to the top.^7^ Interestingly, we also observed an upregulation of *PIGR* expression in a subset of goblet cells after the ingestion of solid food, which suggests that transepithelial transport of IgA could be an unexplored function of mucus secreting cells. The presence of IgA in rabbit cecal goblet cells, as observed previously in the intestine of birds,^39^ could be explained by the binding of IgA to mucins or their transport through goblet cell–mediated passage.^40^ Future research is required to explore the potential contribution of goblet cells to IgA transport across the intestinal epithelium.

Although our results indicate that most transcriptome changes induced by solid food ingestion are cell type specific, we also found a few genes similarly regulated in most epithelial cell types. A striking example is the panepithelial upregulation of *ALDH1A1*, which is involved in epithelial processing of dietary vitamin A into retinoic acid.^41^ Epithelial retinoic acid metabolism was previously shown to be upregulated at the weaning transition in the mouse intestine^42^ and is known to be influenced by the gut microbiota,^41^ notably through the bacterial metabolite butyrate, which was shown to induce *ALDH1A1* expression in human and mouse small intestinal 3D organoids.^43^ In contrast, we found that butyrate did not change the expression of *ALDH1A1* in rabbit cecum organoid cell monolayers, potentially due to differences in culture format, gut segment, or species. Given the role of retinoic acid in tuning intestinal immune responses,^44^ our results suggest that epithelial regulation of vitamin A metabolism at the onset of solid food ingestion may contribute to the “weaning reaction,” which corresponds to a transient remodeling of mucosal immunity essential to program mucosal health.^25^ In our study focusing on the epithelial layer, we found that a prominent feature of this “weaning reaction” was the strong upregulation of ISGs, which was previously shown to be a microbiota-dependent process.^27^ Our study newly shows that this solid food–induced upregulation of ISGs is mostly restricted to crypt-top mature ACs and to BEST4^+^ cells. This observation is in line with previous studies in mice showing that microbial colonization induced the upregulation of ISGs specifically in subsets of mature ACs localized at the tip of epithelial villi in the small intestine.^45^^,^^46^ The solid food–induced upregulation of ISGs coincided with an increased concentration of butyrate and a higher abundance of the butyrate-producing family Lachnospiraceae,^47^ which is probably driven by the introduction of plant-based complex carbohydrates. Accordingly, we found that butyrate strongly increased the expression of the ISG *OASL* in cell monolayers derived from rabbit cecum organoids, which is in agreement with a previous study in chicken cells.^48^ In contrast with the upregulation of ISGs, we found that solid food ingestion reduced the expression of numerous cytokines and antimicrobial peptides in a cell type–specific manner, indicating an overall remodeling of epithelial defense systems. For instance, our data revealed that BEST4^+^ cells are the main producers of the immunomodulating *IL33* alarmin,^49^ which is downregulated after ingestion of solid food. We also confirmed the goblet cell–specific expression of the recently discovered antimicrobial peptide *WFDC2*,^10^ and we newly report its downregulation after the introduction of solid food.

Our results showing that solid food ingestion alters the gene expression of membrane mucins (*MUC1*, *MUC12*, *MUC13*), specifically in absorptive and BEST4^+^, cells highlight the cell types involved in the establishment of the glycocalyx, which was previously shown in mice to be part of an adaptation of the epithelial defense repertoire during weaning.^42^ In addition, we found that several glycosyltransferases involved in the post-translational modification of mucins were regulated by the introduction of solid food predominantly in stem cells and proliferating cells located at the crypt base. This effect may be driven by the changes in the gut microbiota induced by solid food ingestion since a previous study showed that microbial colonization of the mouse intestine similarly changed glycosylation in stem and TA cells.^50^ The mature AC-specific upregulation of the fucosyltransferase *FUT2* induced by the ingestion of solid food could also be driven by microbial signals but also by changes in glucocorticoid levels at the weaning transition, as previously demonstrated in mice.^51^^,^^52^ The solid food–induced upregulation of mucus components secreted by goblet cells (*ZG16*, *TFF2*) could contribute to protect the intestinal epithelium from microorganisms expanding in the gut at the weaning transition.

The remodeling of epithelial defense systems induced by the introduction of solid food coincided with a shift of the transcriptome of cecal epithelial cells characterized by a reduced expression of small intestine-specific genes (*ANPEP*, *APOB*) and a higher expression of large intestine–specific genes (*AQP8*, *CA1*, *SLC26A3*).^5^ The changes in the gut microbiota induced by solid food ingestion could contribute to the acquisition of these large intestine–specific functions because microbial colonization of the rat intestine was previously shown to induce similar effects.^53^ Our experiments in cell monolayers derived from rabbit cecum organoids suggest that the increased production of butyrate by the gut microbiota after the onset of solid food ingestion could contribute to epithelial differentiation. The regional specialization of epithelial cells upon solid food introduction was associated with a strong shift in amino acid and lipid metabolism. Indeed, solid food introduction altered the AC expression of transporters of amino acids whose concentration was reduced in the lumen. Increased utilization of milk-derived amino acids by the microbiota for bacterial growth could lower their availability,^54^ which could explain the lower urea concentration in the plasma and the increased expression of its transporter (*SLC14A2*) in ACs after the introduction of solid food. The effects of solid food ingestion on the expression of lipid handling genes in ACs could also be driven by changes in the gut microbiota, which has previously been shown to regulate lipid homeostasis in the intestinal epithelium during the weaning transition in mice.^27^ Indeed, metabolites produced by the gut microbiota in early life regulate lipid metabolism in epithelial cells.^55^^,^^56^ In contrast, changes in dietary lipid supply can be ruled out, as lipids are mainly derived from maternal milk,^57^ the amount of which was not reduced after the introduction of solid food. Changes in the gut microbiota triggered by solid food ingestion could also contribute to the upregulation of basolateral bile acid exporters (OSTα/β coded by *SCL51A/B* genes) in ACs and to the reduction of the plasmatic concentration of cholesterol, the precursor of bile acids.^27^^,^^58^^,^^59^ In turn, solid food–induced modification of bile acid metabolism could contribute to the maturation of the microbiota, as demonstrated in mice at the suckling-to-weaning transition.^60^ Interestingly, we found that the cytosolic bile acid binding protein (*FABP6*) was specifically expressed and upregulated in BEST4^+^ cells after solid food ingestion, which suggests an uncovered role for these cells in the enterohepatic circulation.^61^

Our study is focused on epithelial cells, whereas major changes are known to occur in intestinal immune cells during the weaning transition, as demonstrated in mice.^25^ Our initial scRNA-seq dataset included some intraepithelial lymphocytes, but their numbers were insufficient to perform reliable analyses. Future studies analyzing the single-cell transcriptome of lamina propria immune cells in our suckling rabbit model ingesting or not solid food are needed to expand our understanding of the gut barrier maturation during the weaning transition. Indeed, the transcriptome changes that we observed in intestinal epithelial cells after solid food ingestion suggest alterations in the crosstalk with immune cells, particularly in relation to interferon and cytokine signaling. Another limitation of our study is related to its restriction to epithelial cells isolated from the cecum, which we chose because this large intestine region harbors a dense microbial population that is highly responsive to dietary changes at the suckling-to-weaning transition.^24^ Additional studies examining single-cell transcriptome changes induced by solid food ingestion in other regions of the gut, such as the jejunum, may also be relevant to explore metabolic and immune modulations. Furthermore, previous mouse studies have shown that microbiota changes are directly involved in epithelial bulk transcriptome modifications at the weaning transition,^23^^,^^27^ while our study performed at the single-cell level did not evaluate this causal role. The recent development of intestinal organoids that recapitulate the cellular diversity of the epithelium in vitro, including in rabbits,^62^ will be useful in future scRNA-seq studies aiming at evaluating the cell type–specific transcriptome changes induced by gut bacteria or metabolites modified at the weaning transition. Although we are not aware of sex differences in gut maturation, our results should also be confirmed in females, as all experiments were performed in male rabbits only in order to reduce inter-individual variability because of the small sample size (n = 4 per group). Due to differences in weaning patterns and dietary intake between humans and rabbits, extrapolation of our results to human intestinal development should be made with caution.

In conclusion, our study provides the first single-cell transcriptomic atlas of the rabbit intestinal epithelium and significantly expands the understanding of cellular diversity in the mammalian intestine. We highlighted the homology between rabbit and human intestinal epithelial cells, such as BEST4^+^ cells, supporting the suitability of the rabbit as a model for gastrointestinal research. In addition, we uncovered cell type–specific transcriptome modifications driven by solid food ingestion at the suckling-to-weaning transition, highlighting changes in epithelial defense mechanisms and metabolic processes. Our organoid experiments suggest that the increased production of butyrate by the gut microbiota after the onset of solid food ingestion may contribute to epithelial maturation. These findings contribute to a broader understanding of the postnatal maturation of the gut barrier in mammals. Further studies are required to examine the functional and long-term consequences of the transcriptomic changes induced by the ingestion of solid food in each epithelial cell type.

## Material and Methods

### Animal Experiments

The experiments were performed at the PECTOUL experimental facility (GenPhySE, INRAE, Toulouse, France). The handling of rabbits followed the recommendations outlined by the European Union's regulations for the protection of animals used in scientific research (2010/63/EU), and was consistent with the French legislation (NOR: AGRG1238753A 2013). This project received approval from the local ethics committee “Comité d’éthique en expérimentation animale SCIENCE ET SANTE ANIMALES” N°115 (SSA_2022_012 and SSA_2024_004V2). Multiparous dams (n = 4) were housed individually in wire cages (61 × 68 × 50 cm) equipped with a closed nest (39 × 27 × 50 cm). The litter size was limited to 10 pups per litter. From postnatal day 4 (PND4), pups were placed in a new cage, adjacent to their mother’s cage. At PND12, litter sizes were reduced to 6 pups in order to maximize milk ingestion. Pups from each litter were separated in 2 cages on either side of their mother’s cage (3 pups/cage) to form 2 groups (Figure 1A). In the first group (milk), the pups were exclusively suckling. In the second group (milk+solid), the pups were suckling while having ad libitum access to commercial solid food pellets (StabiGreen; Terrya). During the whole experiment, the dam and the pups were placed once a day for 5–10 minutes in the nest of the dam’s cage for suckling before returning to their respective cages. Coprophagia was prevented by removing feces dropped by the mother in the nest after each suckling. Individual milk intake was quantified daily (from PND12 onward) by weighing pups before and after suckling. Solid feed intake was measured daily at the cage level (3 pups) by weighing the feeder. The experiment was repeated a second time independently with n = 6 litters in order to collect samples for quantitative polymerase chain reaction (qPCR) analysis, RNA in situ hybridization, immunohistochemistry, electron microscopy, and calprotectin measurements. All other measurements were performed on samples collected during the first experiment.

### Sample Collection

One male pup per litter and per group (milk or milk+solid) was sacrificed after suckling at PND24 or PND25 by electronarcosis followed by exsanguination (Figure 1*A*). In the first experiment with 4 litters, the samples were collected from 4 pups from the milk group and 4 pups from the milk+solid group. In the second experiment with 6 litters, the samples were collected from 6 pups from the milk group and 6 pups from the milk+solid group. Due to the small sample size, the experiment was performed in males only in order to reduce the potential sex-related variability. Blood collected in EDTA tubes was centrifuged (1000 *g*, 10 minutes, 4 °C) and plasma was stored at –20 °C. The cecum with its content and appendix was isolated and weighed. The content of the cecum was collected and kept at –80 °C until microbiota and metabolome analysis. A fragment of cecal tissue was collected and placed in cold phosphate-buffered saline (PBS) without Ca^2+^/Mg^2+^ (Thermo Fisher Scientific; cat#10010-015) for epithelial cell isolation. Other sections of cecal tissue were fixed in (1) Carnoy solution (60% ethanol, 30% chloroform, 10% glacial acetic acid) for 3 hours before transfer in 70% ethanol (samples used for Alcian Blue and Periodic Acid of Schiff staining), (2) 10% neutral-buffered formalin for 24 hours before transfer in 70% ethanol (samples used for immunohistochemistry and RNA in situ hybridization), or (3) 0.1 M Sörensen phosphate buffer (pH 7.4) with 2% glutaraldehyde at 4 °C (samples used for electron microscopy). Sections of the duodenum, jejunum, ileum, cecum and colon were snapped frozen in liquid nitrogen and stored at –80 °C until qPCR gene expression analysis.

### Cecal Epithelial Cell Isolation

Cecal tissue was opened longitudinally and washed with cold PBS to remove all content. The tissue was minced into 1 cm^2^ sections and washed with cold PBS. Tissue segments were transferred to 5 mL of a prewarmed (37 °C) digestion solution prepared in HBSS without Ca^2+^/Mg^2+^ (Thermo Fisher Scientific; cat#14175095) and supplemented with 5 mM EDTA (Thermo Fisher Scientific; cat#AM9260G) and 1 mM DTT (Sigma-Aldrich; cat#10197777001). After incubation (20 minutes at 37 °C under slow agitation at 15 rpm), epithelial crypts were detached by vigorous manual shaking for 1 minute. The crypt solution was then filtered (100 μm) before centrifugation (300 *g* for 5 minutes at 4 °C). The crypt pellet was resuspended in 10 mL of pre-warmed dissociation solution containing TrypLE (Thermo Fisher Scientific; cat#1205036) supplemented with 1 mg/mL DNAse I (Sigma-Aldrich; cat#10104159001), 5 mM MgCl_2_ (Sigma-Aldrich; cat#M1028), 10 μM Y27632 (StemCell Technologies; cat#72304) and the solution was distributed in two 50 mL conical tubes (5 mL/tube). Cells were incubated for 10 minutes at 37 °C under gentle agitation at 15 rpm before homogenization by vortexing (3 seconds). This step was repeated and followed by 2 successive filtrations (70 μm and 40 μm). Digestion was stopped by adding 45 mL of cold PBS to the cells. After centrifugation (300 *g* for 5 minutes at 4 °C), the cells were resuspended in 5 mL FACS buffer (PBS supplemented with 3% fetal bovine serum [Thermo Fisher Scientific; cat#10270-106], 2 mM EDTA, and 10 μM Y27632). Cell concentration was measured using an automated cell counter Countess 3 (Thermo Fisher Scientific; cat#AMQAX2000).

### Cell Preparation for Single-Cell Sequencing

Cells (2×10^6^) were centrifuged (300 *g* for 5 minutes at 4 °C) and resuspended in 1 mL of PBS supplemented with 10 μM Y27632. This step was repeated twice. Dead cells were stained with the LIVE/DEAD Fixable Violet Dead Cell Stain Kit (Thermo Fisher Scientific; cat#L34963), according to the manufacturer’s instructions. After 30 minutes of incubation (4 °C, protected from light), cells were centrifuged (300 *g* for 5 minutes at 4 °C) and resuspended in 1 mL FACS buffer. This step was repeated once. Cells were filtered (40 μM) and sorted (10^5^ live and single cells) in a 1.5 mL tube containing 10 μL of PBS supplemented with 10 μM Y27632 by using a BD Influx cell sorter instrument with a 100 μm nozzle, under 20 psi at the I2MC Cytometry and Cell sorting TRI platform (Toulouse, France). After centrifugation (300 *g* for 5 minutes at 4 °C), cells were resuspended in 100 μL PBS, counted manually and their viability was verified by trypan blue staining.

### Single-Cell Sequencing

For scRNA-seq, approximately 10,000 cells per sample were used for encapsulation into droplets using Chromium Next GEM Single-cell 3′ Reagent Kits v3.1 according to manufacturer’s protocol (10x Genomics CG000315 Rev E user guide). Briefly, after generation of GEMs using Next GEM Chip G, GEMs were reverse transcribed in a C1000 Touch Thermal Cycler (Bio-Rad) programmed at 53 °C for 45 minutes, 85 °C for 5 minutes, and held at 4 °C. After reverse transcription, single-cell droplets were broken and cDNA was isolated and cleaned with Cleanup Mix containing DynaBeads (Thermo Fisher Scientific). cDNA was then amplified with a C1000 Touch Thermal Cycler programmed at 98 °C for 3 minutes; 12 cycles of 98 °C for 15 seconds, 63 °C for 20 seconds, and 72 °C for 1 minute; and 72 °C for 1 minute; and held at 4 °C. Subsequently, approximately 250 ng of amplified cDNA was fragmented, end-repaired, A-tailed, index adaptor ligated, and cleaned with cleanup mix containing SPRIselect Reagent Kit (Beckman Coulter; cat#B23317) in between steps. Postligation product was amplified and indexed with a C1000 Touch Thermal Cycler programmed at 98 °C for 45 seconds; 11 cycles of 98 °C for 20 seconds, 54 °C for 30 seconds, and 72 °C for 20 seconds; and 72 °C for 1 minute; and held at 4 °C. The sequencing-ready libraries were cleaned up with SPRIselect beads. 10x libraries were pooled and charged with 1% PhiX on 1 S1 lane of the NovaSeq 6000 instrument (Illumina) using the NovaSeq 6000 S1 Reagent Kit v1.5 (100 cycles), and the following sequencing parameters: 28 bp read 1 – 10 bp index 1 (i7) – 10 bp index 1 (i5) – 150 bp read 2. The S1 lane generated a total of 810 × 10^6^ raw reads.

### scRNA-seq Preprocessing, Filtering, Normalization, and Clustering

Cell Ranger Software (version 7.1.0, 10x Genomics) was used to align and quantify raw sequencing data using the rabbit reference genome (GCF_009806435.1_UM_NZW_1.0). A custom reference file was created using the Cell Ranger mkgtf command with “--attribute=gene_biotype:protein_coding and --attribute=gene_biotype:lncRNA” parameters. The Cell Ranger mkref and count commands were used with default parameters.

**Figure 13:**
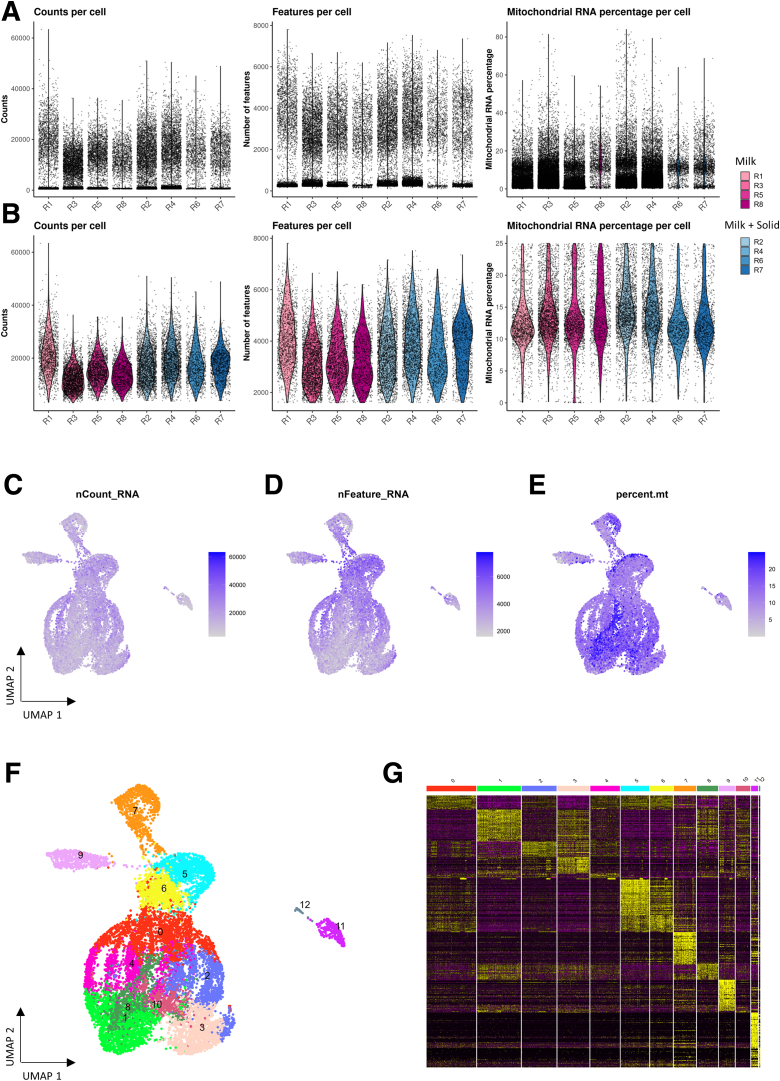
**Quality controls of single-cell transcriptomics.** (*A*) Counts, number of expressed genes, and percentage of mitochondrial gene reads per cell and rabbit before filtering. (*B*) Counts, number of expressed genes, and percentage of mitochondrial gene reads per cell and rabbit after filtering. (*C*) UMAP of cells colored by the total counts per cell. (*D*) UMAP colored by the number of expressed genes. (*E*) UMAP colored by the percentage of mitochondrial gene reads. (*F*) UMAP colored by clusters. (*G*) Expression of the top 50 genes with the highest average log2(fold change) of each cluster. For a given cluster, markers were ordered by decreasing log2(fold change) of the expression between this cluster and the other clusters. Cell clusters are indicated by numbers and colored bars on the top.

Using R software (version 4.2.1; R Foundation for Statistical Computing), the Seurat (version 4.3.0) pipeline^63^ was run for data preprocessing and analysis. SeuratObjects were generated for each rabbit (n = 8) and merged. Cells with <1600 or more than 55,000 expressed genes were filtered out (Figure 13*A–E*). Similarly, cells with a number of counts below 1500, with a percentage of mitochondrial RNA above 25% or expressing more than 0.1% of counts from hematopoietic cell genes (*CD44*, *PTPRC*, *CD48*) were filtered out. The resulting data were normalized via the *NormalizeData* function of Seurat, with the *LogNormalize* method. The top 2000 variable features were then extracted (based on a mean-variance trend as implemented in the *FindVariableFeatures* function of Seurat). After scaling data to unit variance, dimensionality reduction was carried out with principal component (PC) analysis (50 PCs). Cell clustering was performed based on retained PCs and using the Leiden algorithm on a cell similarity graph, with a 0.5 resolution. Finally, clusters were visualized using the nonlinear reduction dimensionality UMAP performed on PC analysis reduction (30 PCs) (Figure 13*F*).

### Cell Type Assignment

#### Marker genes

The marker gene list for each cluster was obtained using a Wilcoxon test as implemented in the Seurat function *FindMarkers* (Figure 13*G*). A gene was declared a marker if its adjusted *P < .*05 (Bonferroni correction for multiple testing). The test results were further filtered to ensure a minimum log2-fold change of 0.25 between the tested cluster and the others. Only genes expressed in at least 25% of cells and overexpressed in the tested cluster (compared to the others) were considered for this analysis. Cell types were then manually assigned to clusters according to found markers, based on a comparison with known cell-type markers.^5^^,^^10^^,^^64^^,^^65^

#### Assessment of cluster validity with cell cycle score and crypt axis gene score

The cell cycle score was used to assign phases of the cell cycle to individual cells and assess the consistency between manually assigned cell types and expected cell cycle phase. The cell cycle score was computed with the Seurat function *CellCycleScoring*. In addition, the crypt axis gene score of each cell was calculated via the *AddModulesScore* function by averaging the expression of genes previously defined as expressed in epithelial cells located at the crypt top (*PLAC8*, *CEACAM1*, *TSPAN1*, *DHRS9*, *RHOC*, *PKIB*, *HPGD*).^10^

#### Pseudo-time analysis

**Figure 14:**
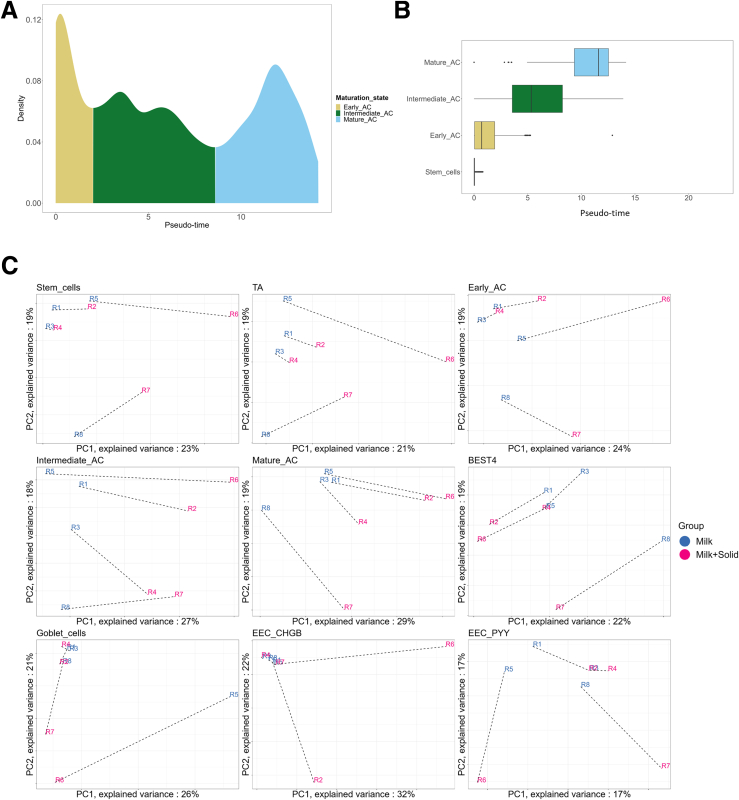
**Pseudo-time and PC analysis.** (*A*) Pseudo-time distribution of ACs. The area under the curve is colored with respect to chosen AC subset borders. (*B*) Pseudo-time distribution in stem cells and AC subsets. (*C*) PC analysis performed on pseudo-bulk data for each epithelial cell type. Individual samples are represented by the rabbit (R) identifier. The dotted lines connect rabbit littermates.

Considering that AC subsets are distinguishable from each other based on their differentiation states, we used a pseudo-time analysis to create AC subgroups with the monocle3 package (version 3.1). The trajectory of cell types was obtained using the *learn_graph* function on the previously generated UMAP and the pseudo-time of each cluster was calculated based on their projection on the trajectory using the *order_cells* function. The trajectory root was set to be the stem cell cluster. Subsequently, 3 cell subgroups of AC were delineated based on the pseudo-time distribution, which was found to be trimodal: cells with a pseudo-time below 2.0 were classified as “Early AC,” those with a pseudo-time between 2.0 and 8.6 were classified as “Intermediate AC,” and cells with a pseudo-time above 8.6 were classified as “Mature AC” (Figure 14*A* and *B*). Marker extraction was performed for each assigned cell type, similarly to what was performed for cluster markers and as described in the “Marker genes” section (Supplementary Table 1).

#### Automatic assignation of cell types

To validate our manual annotation, we performed an automatic annotation based on the transfer of labels of a reference to the rabbit cecum epithelial cells.^66^ Human epithelial cells from the large intestine^64^ were used as a reference. First, PC analsyis was performed on the reference dataset to reduce its dimension to the first 30 PCs. Then, *FindTransferAnchors* was used to find similar cells between the rabbit and human datasets, called “anchors.” These anchors were then used by the *MapQuery* function to map the rabbit cecum epithelial cells onto the human epithelial cell space. The reference annotation was then transferred from the reference to the rabbit data and visualized on the UMAP. The results of *MapQuery* were also used in the *MappingScore* function to attribute a score to each rabbit cell. Roughly, this score measures how a cell neighborhood is affected by a mapping to and then back from the reference (a higher score corresponds to a more similar neighborhood).

#### Biological pathways enrichment

Biological enrichment analysis was performed on marker genes of the cell types using the *enrichGO* function from the *clusterProfiler* (version 4.6.1) package,^67^ with all expressed genes as the reference background (Supplementary Table 2). The enrichment analysis was carried out using the *Homo sapiens* database because of the absence of an *Oryctolagus cuniculus* database. Redundancy of results was reduced by using the *simplify* function from the *clusterProfiler* package. Terms with a semantic similarity over 0.7 were deleted and only representative terms (terms with the smallest *P* value) were kept within each group of term. *P* values were corrected for multiple testing using the Benjamini-Hochberg (BH) procedure,^68^ and pathways were considered enriched if their corresponding adjusted *P* value was <.05.

#### Differential analysis of gene expression

The pseudo-count data were derived by summing the counts of each gene across cells of the same type for each rabbit. This step is considered essential as it has been shown that performing the differential analysis on pseudo-bulk data yields more robust results, reducing the risk of type I errors compared to analyzing scRNA-seq data directly.^69^^,^^70^ The whole analysis was performed independently in each cell type. Pseudo-counts were normalized using the “TMM” method of e*dgeR*.^71^ A PC analysis was conducted on log2-transformed pseudo-counts for quality control, revealing a possible important impact of the litter on gene expression (Figure 14C). This was thus accounted for in the differential analysis. Differential expression analysis was performed using a Negative Binomial generalized linear model as implemented in *edgeR*. More precisely, each gene expression was modeled with an additive effect of both the group and the litter, the latter being used as a blocking variable. *P value*s were obtained with a log-likelihood ratio test of the group effect. Adjusted *P* values were obtained with the BH procedure and genes were considered differentially expressed if their corresponding adjusted *P* value was < 0.05 (Supplementary Table 3). Differentially expressed genes were subjected to an enrichment analysis as described in the *“*Biological pathways enrichment” section (Supplementary Table 4).

### Microbiota Composition

The microbiota composition was analyzed as described previously.^24^ Briefly, DNA was extracted from 50 mg of cecal content with the Quick-DNA Fecal/Soil Microbe DNA Miniprep Kit (Zymo Research; cat#D6010). The V3-V4 region of the 16S gene was amplified by PCR and amplicons were sequenced by MiSeq Illumina Sequencing. Bioinformatic analyses were performed with the FROGS pipeline (v.4.0.1) according to the guidelines.^72^ Taxa representing more than 0.5% of the relative abundance in at least 1 group were considered for analysis (Supplementary Table 5), as it was previously shown that taxa below this threshold were not accurately quantified.^73^

### Metabolomics

The metabolome was analyzed in 50 mg of cecal content by using nuclear magnetic resonance (NMR)-based metabolomics, at the MetaboHUBMetaToul-AXIOM metabolomics platform (GenotToul, Toulouse, France), as described previously.^24^ The relative concentration of each metabolite was expressed relatively to the mean concentration measured in the milk group.

### Plasma Biochemistry

The Clinical Chemistry Analyzer Pentra C400 (Horiba Medical) was used at the Anexplo Phenotyping platform (GenoToul) to measure plasmatic concentrations of cholesterol, high-density lipoprotein, low-density lipoprotein, glucose, triglycerides, free fatty acids, and urea.

### Calprotectin Assay

Cecal epithelial cells isolated as described above were lysed in RIPA buffer (Thermo Fisher Scientific; cat#89901) supplemented with cOmplete protease inhibitor cocktail (Roche; cat#11697498001) by using stainless steel beads and a TissueLyser II (Qiagen) operating at 30 Hz for 3 minutes. Lysates were centrifuged (12,000 *g*, 10 minutes, 4 °C) and stored at –80 °C until analysis. Calprotectin was quantified in undiluted epithelial cell lysates by using a rabbit-specific ELISA kit (Clinisciences; cat#MBS2601529-48), following the manufacturer instructions. Protein concentration in epithelial cell lysates diluted 1:2 (v/v) in NaCl 0.9% was measured with Pierce Bradford Plus Protein Assay Kits (Thermo Fisher Scientific; cat#23236). Calprotectin concentration was normalized to the protein level of each sample.

### Histology

Transversal sections of cecal tissue with luminal content fixed in Carnoy’s solution were embedded in paraffin and stained by Alcian blue and Periodic Acid Schiff at the histology platform Anexplo (GenoToul). Slides were digitalized before measurement of the crypt depth and of the number of goblet cells per crypt with the CaseViewer 2.3 software (3DHISTECH). Formalin-fixed, paraffin-embedded transversal sections of cecal tissues were cut into 4 μm sections and adhered to Superfrost-Plus charged microscope slides (Thermo Fisher Scientific) before being used for RNA in situ hybridization and immunohistochemical staining.

#### RNA in situ hybridization

The RNAscope 2.5 HD Reagent Kit – RED (Advanced Cell Diagnostics; cat#322350) and the RNAscope Multiplex Fluorescent Reagent Kit v2 (Advanced Cell Diagnostics; cat#323100) were used with rabbit custom probes targeting *SPINK4* (Advanced Cell Diagnostics; cat#1564251-C1, RNAscope Probe-Oc-SPINK4-C1 or cat#1564251-C2, RNAscope Probe-Oc-SPINK4-C2), *BEST4* (Advanced Cell Diagnostics; cat#1564261-C1, RNAscope Probe-Oc-BEST4-C1), *PIGR* (Advanced Cell Diagnostics; cat#1003001-C1, RNAscope Probe-Oc-PIGR-C1), or *CFTR* (Advanced Cell Diagnostics; cat#497241-C2, RNAscope Probe-Oc-CFTR-C2). Negative and positive control slides were respectively hybridized with the RNAscope Negative Control Probe-DapB (Advanced Cell Diagnostics; cat#310043) and RNAscope Probe-Oc-GAPDH-No-XHs (Advanced Cell Diagnostics; cat#469461) or RNAscope Probe-Oc-POLR2A (Advanced Cell Diagnostics; cat#410571).

The chromogenic assay (*SPINK4*) was performed as described before with minor modifications (Palmer et al).^74^ Slides were incubated 30 minutes at 60 °C to enhance tissue adherence. Slides were deparaffinized using xylene and rehydrated through a series of graded ethanol washes. Slides were treated with hydrogen peroxide to block endogenous peroxidase activity, followed by epitope unmasking using a boiling target retrieval solution. Hydrophobic barriers were drawn around the tissues to contain reagents. Unless stated otherwise, all incubations were performed at 40 °C in a HybEZ hybridization oven followed by a 1× Wash Buffer wash. Slides were incubated with the Protease Plus for 15 minutes to break down RNA-associated proteins. Probes were then applied to each slide and incubated for 2 hours. The amplification steps were carried out sequentially using AMP1, AMP2, AMP3, AMP4, AMP5, and AMP6, with varying incubation times (30, 15, 30, 15, 30, and 15 minutes) and temperatures (40 °C for AMP1–4, and room temperature [RT] for AMP5 and AMP6). Chromogenic detection was performed using a 1:60 dilution of RED-A:RED-B, followed by a counterstaining with Gill’s Hematoxylin I (American Master Tech Scientific; cat#HXGHE1LT, diluted 1:1 in dH_2_O). Slides were immediately air-dried for 20 minutes at RT, after which 2 drops of Vecta Mount (Vector Laboratories; cat#H-5700-60) were applied. Coverslips (#1 thickness) (Fisherbrand; cat#12-545-F) were mounted, and the slides were left to dry for 20 minutes at RT.

The fluorescent in situ hybridization singleplex staining (*PIGR* and *BEST4*) protocol is available on protocols.io (dx.doi.org/10.17504/protocols.io.j8nlk99q1v5r/v1). The assay was performed identically to the chromogenic assay for the pretreatment and the hybridization steps. The amplification was performed with AMP1 (30 minutes), AMP2 (30 minutes), and AMP3 (15 minutes). Detection was performed with HRP-C1 for 15 minutes and washed. The Opal 570 Reagent fluorophore (Akoya Biosciences; cat#FP1488001KT, dilution 1:750 in RNAscope Multiplex TSA Buffer [Advanced Cell Diagnostics; cat#322809]) was incubated on the slides for 30 minutes. After the wash, the HRP blocker was added to the slides and incubated for 15 minutes. Incubations were performed at 40 °C in a HybEZ oven, followed by a wash performed twice with 1X Wash Buffer for 2 minutes at RT. All the slides were incubated with DAPI (Advanced Cell Diagnostics; cat#323108) for 30 seconds at RT and mounted with 2 drops of ProLong Gold Antifade reagent (Invitrogen; cat#P36930) and covered with #1.5 thickness cover glass (Fisherbrand; cat#12-545-F).

The fluorescent in situ hybridization duplex staining (C1-*PIGR* and C2-*SPINK4* or C1-*BEST4* and C2-*SPINK4* or C1-*BEST4* and C2-*CFTR*) protocol is available on protocols.io (dx.doi.org/10.17504/protocols.io.14egn99kql5d/v1). The probe solution applied to each slide contained the C2 probes diluted in the C1 probe solution (1:50). After the application of the Opal 570 Reagent fluorophore (Akoya Biosciences; cat#FP1488001KT, dilution 1:750 in RNAscope Multiplex TSA Buffer [Advanced Cell Diagnostics; cat#322809]) and the incubation with HRP blocker, the detection of the C2 probes was performed with HRP-C2 for 15 minutes and washed. The Opal 690 Reagent fluorophore (PerkinElmer; cat#FP1497A, dilution 1:1200 in RNAscope Multiplex TSA Buffer [Advanced Cell Diagnostics; cat#322809]) was incubated on the slides for 30 minutes. After the wash, the HRP blocker was added to the slides and incubated for 15 minutes.

#### Chromogenic immunohistochemistry

The immunohistochemistry assay was performed as described before.^75^ Briefly, slides were incubated for 20 minutes at 60 °C, deparaffinized in xylene, and rehydrated with ethanol and distilled water using a histological automaton (Leica Biosystems; cat#ST5020). Antigen retrieval was performed by submerging the slides in preheated distilled water, followed by incubation in 1X sodium citrate solution for 15 minutes at 95 °C. Hydrophobic barriers were drawn around the sections, and slides were incubated with Dual Endogenous Enzyme Block (Dako; cat#S2003) for 10 minutes at RT, Protein Block (Dako; cat#X0909) for 20 minutes at RT, and primary antibody (Goat Anti-Rabbit IgA; Abcam Limited; cat#ab97186, 1:3000; Mouse anti-KI67; BD Biosciences; cat#AB_393778; 1:40; dilution in 1% bovine serum albumin PBS, 4 °C overnight) sequentially. Slides were then incubated with a secondary antibody (ImmPRESS HRP Anti-Goat Ig, Vector; cat#MP-7405; or HRP Labelled Polymer Anti-Mouse, Dako; cat#K400111-2) for 30 minutes at RT, followed by DAB chromogen for 7 minutes at RT in the dark, and counterstained with 25% diluted Gill’s hematoxylin (American Master Tech Scientific; cat#HXGHE1LT) for 1 minute at RT. Slide washes were performed after each incubation using a 0.05% PBS-Tween solution. Slides were dehydrated in ethanol and Propar clearant (Anatech; cat#510) sequentially, mounted with Refrax Mounting Medium (Anatech; cat#711), and covered with #1 thickness cover glass (Fisherbrand; cat#12-545-F) using a histological automaton (Leica Biosystems; cat#ST5020). Negative controls were treated with 1% bovine serum albumin in PBS without the primary or secondary antibodies.

### Electron Microscopy

Electron microscopy analyses were performed in CMEAB (Toulouse, France). For transmission electron microscopy, following fixation, samples were washed overnight in 0.2 M Sörensen phosphate buffer (pH 7.4). Postfixation was carried out at RT for 1 hour in 0.05 M Sörensen phosphate buffer (pH 7.4) with 1% OsO_4_ and 0.25 M glucose. Dehydration was performed using graded ethanol series at RT, up to 70%. From then, the tissues were embedded in Embed 812 resin (Electron Microscopy Sciences) using a Leica EM AMW automated microwave tissue processor for electron microscopy. Once poylymerized, the samples were sliced into ultrathin sections (70 nm) using an Ultracut Reichert Jung ultramicrotome and mounted onto 100-mesh Formvar-coated copper grids. Sections were then stained with 3% uranyl acetate in 50% ethanol and Reynold’s lead citrate. Examinations were conducted on a transmission electron microscope (Hitachi HT7700) at an accelerating voltage of 80 kV. For scanning electron microscopy, after washing the sample in water, dehydration was performed through a graded ethanol series, up to 100% ethanol. Critical point drying was carried out with a Leica EM CPD 300. Dried samples were then coated with a 6 nm layer of platinum using a Leica EM MED020. SEM imaging was performed using a FEG FEI Quanta 250 scanning electron microscope at an accelerating voltage of 5 kV.

### Culture of Rabbit Cecum Organoids in 3D

Cecum organoids derived from suckling rabbits (18 days old) were obtained from our in-house biobank.^62^ Briefly, cryopreserved cecum epithelial crypts kept in liquid nitrogen were thawed at 37 °C, centrifuged (500 *g*, 4 °C, 5 minutes) and seeded in Matrigel (Corning; cat#354234) in a prewarmed 48-well plate (25 μL/well). Organoid growth culture medium containing IntestiCult Organoid Growth Medium (Human) (StemCell Technologies; cat#6010) supplemented with 1% penicillin-streptomycin (Sigma-Aldrich; cat#P4333) and 100 μg/mL Primocin (InvivoGen; cat#ant-pm-05) was added (250 μL/well). Organoids were cultured at 37 °C with 5% CO_2_. Seven days after seeding, organoids in Matrigel were washed in PBS (Thermo Fisher Scientific; cat#10010015) and homogenized by pipetting in warm TrypLE (Thermo Fisher Scientific; cat#12605-010) before incubation for 10–15 minutes at 37 °C. Digestion was stopped by adding cold complete DMEM containing DMEM (Thermo Fisher Scientific; cat#31966047) supplemented with 10% fetal bovine serum (FBS, Thermo Fisher Scientific; cat#10270-106) and 1% penicillin-streptomycin. Cells were centrifuged (500 g, 4 °C, 5 minutes) and counted using a Countess 3 Automated Cell Counter (Thermo Fisher Scientific; cat#16842556). Organoid cells were seeded for expansion in 3D in Matrigel:complete DMEM (v/v: 2:1) in prewarmed 24-well plates (3000 cells/50 μL/well) and organoid culture medium was added (500 μL/well) and replaced every 2–3 days. Organoids were used to seed cell monolayers 7 days after seeding. Experiments were repeated with organoid cells derived from 5 rabbits.

### Culture of 2D Cell Monolayers Derived From Rabbit Cecum Organoids

Cell culture inserts for 24-well plates (Corning; cat#353095) were coated with 50 μg/mL Collagen type IV from human placenta (Sigma-Aldrich; cat#C5533) for 2 h at 37 °C (150 μL/well). The coating solution was removed and the inserts were dried for 10 minutes by opening the plate lid under the cell culture cabinet. Organoids were dissociated and cells were counted and centrifuged as described above. Cells were resuspended in organoid growth culture medium supplemented with 20% FBS and 10 μM Y27632 (ATCC; cat#ACS-3030) before seeding in inserts (10^5^ cells/insert). The same medium was used at the basal side. Cells were incubated at 37 °C, 5% CO_2_. Three days after seeding, the apical and basal medium was replaced by organoid differentiation medium containing IntestiCult Organoid Differentiation Medium (Human) (StemCell Technologies; cat#100-0214) supplemented with 1% penicillin-streptomycin (Sigma-Aldrich; cat#P4333), 100 μg/mL Primocin (InvivoGen; cat#ant-pm-05), and 5 μM DAPT (Thermo Fisher Scientific; cat#J65864.MA). Four days after seeding, the apical medium was replaced by organoid differentiation culture medium supplemented or not with 5 mM sodium butyrate (Sigma-Aldrich; cat#B5887). The basal medium was replaced with organoid differentiation culture medium. Six days after seeding, cells were lyzed in 300 μL TriReagent (Ozyme; cat#ZR2050-1-200) and kept at –80 °C until RNA purification.

### Gene Expression Analysis by qPCR

RNA was purified from organoid cells by using the Direct-zol RNA Microprep kit (Zymo Research; cat#R2062) and from intestinal tissue sections by using the Direct-zol RNA Miniprep kit (Zymo Research; cat#R2050), following the manufacturer’s instructions. RNA was eluted in RNase-free water (15 μL for organoids, 50 μL for intestinal tissue sections) and quantified with a NanoDrop 8000 spectrophotometer (Thermo Fisher Scientific). RNA (500 ng for organoid cells, 1 μg for intestinal tissue sections) were reverse transcribed to cDNA by using the GoScript Reverse Transcription Mix, Random primer (Promega; cat#A2801), following the manufacturer instructions. Gene expression was analyzed by real-time qPCR using QuantStudio 6 Flex Real-Time PCR System (Thermo Fisher Scientific) or Biomark microfluidic system using 96.96 Dynamic Arrays IFC for gene expression (Fluidigm) according to the manufacturer’s recommendations. The sequences of the primers used are presented in Supplementary Table 6. Data were normalized to the stably expressed gene *TOP1* (organoid cells) or *ATP5B* (intestinal tissue sections) and analyzed with the 2^-ΔCt^ method.

### Statistical Analyses of Microbiota, Metabolites, Calprotectin, and qPCR Data

Statistical analyses of the log-transformed relative abundances of bacterial taxa and metabolite concentrations (cecum or plasma), calprotectin concentration in epithelial cells and gene expression measured by qPCR in intestinal tissue segments or organoids were performed with R software (version 4.2.1). Linear mixed models with the group (milk or milk+solid) as a fixed effect and the litter as a random effect were estimated to analyze microbiota, metabolite and calprotectin data. Linear mixed models with the gut segment (duodenum, jejunum, ileum, cecum, or colon) as a fixed effect and the rabbit as a random effect were estimated to analyze gene expression measured by qPCR in intestinal tissue sections. Linear mixed models with the treatment (control or butyrate) as a fixed effect and the rabbit from which organoids were derived and the experimental batch as random effects were estimated to analyze gene expression measured by qPCR in cell monolayers derived from organoids. *P* values were corrected for multiple testing using the BH procedure. The significance of the group effect was tested and results were considered significant if their corresponding adjusted *P* value was <.05.
